# Screening and Validation of Independent Predictors of Poor Survival in Pancreatic Cancer

**DOI:** 10.3389/pore.2021.1609868

**Published:** 2021-07-12

**Authors:** Shui Liu, Yan Cai, E. Changyong, Jiyao Sheng, Xuewen Zhang

**Affiliations:** ^1^ Department of Hepatobiliary and Pancreatic Surgery, The Second Hospital of Jilin University, Jilin University, Changchun, China; ^2^ Hospital of Stomatology, Jilin University, Changchun, China; ^3^ Department of Hepatobiliary and Pancreatic Surgery, China-Japan Union Hospital of Jilin University, Jilin University, Changchun, China

**Keywords:** prognosis, pancreatic cancer, the cancer genome atlas, tetraspanin-1, erb-b2 receptor tyrosine kinase 3, gene expression omnibus

## Abstract

Pancreatic cancer is a digestive system malignant tumor with high mortality and poor prognosis, but the mechanisms of progression remain unclear in pancreatic cancer. It’s necessary to identify the hub genes in pancreatic cancer and explore the novel potential predictors in the prognosis of pancreatic cancer. We downloaded two mRNA expression profiles from Gene Expression Omnibus and The Cancer Genome Atlas Pancreatic Cancer (TCGA-PAAD) datasets to screen the commonly differentially expressed genes in pancreatic cancer by limma package in R. Subsequently, measurement of the functional similarity among the 38 DEGs in common was performed to identify the hub genes using GOSemSim package. Then, survival analysis and Cox regression were applied to explore prognosis-related hub genes using the survival package. Statistics analysis by two-tailed Student’s t-test or one-way based on TCGA-PAAD datasets and qPCR detection in clinical samples were performed to explore the correlations between expression of hub genes in pancreatic cancer tissues and clinical parameters. Based on integrated analysis of TCGA and GEO datasets, we screened 38 DEGs in common, which were all up-regulated. The functional similarity results showed that 10 DEGs including TSPAN1, MSLN, C1orf116, PKP3, CEACAM6, BAIAP2L1, PPL, RAB25, ERBB3, and AP1M2 in the DEGs in common, which had the higher average functional similarity, were considered as the hub genes. Survival analysis results and Cox regression analysis showed that TSPAN1, CEACAM6, as well as ERBB3 were all associated with poor overall survival of PC. qPCR results showed that the expression levels of TSPAN1 and ERBB3 were significantly upregulated in the PC tissues. The statistical analysis results revealed that TSPAN1 expression correlated significantly with histologic grade, T stage, clinical stage, and vital status by two-tailed Student’s t-test or one-way ANOVA; ERBB3 expression correlated significantly with T stage, clinical stage, and vital status by two-tailed Student’s t-test or one-way ANOVA. We found that TSPAN1 and ERBB3 could be independent predictors of poor survival in pancreatic cancer.

## Introduction

Pancreatic cancer (PC) is a common digestive system malignant tumor that is characterized by high mortality and poor prognosis. There are more than 458,918 estimated new cases and 432,242 estimated death cases every year around the world in 2018 [1]. Due to its high malignancy, the 5-years survival rate of PC patients is only 10% [2]. Identification of new independent prognostic biomarkers is still of great significance for patients with PC for improving treatment and prognosis of PC patients.

With the advent of the era of big data, a variety of tumor-related public databases have sprung up, which provides a large amount of genomic data and its corresponding clinical data for oncology basic medicine and translational medicine. Over the last decades, public databases, including the well-known The Cancer Genome Atlas (TCGA) database, Gene Expression Omnibus (GEO) database, etc., have been widely applied in screening of the molecular mechanisms of PC, which could provide powerful support for identification of effective and accurate prognosis predictors of PC patients [3, 4].

**FIGURE 1:**
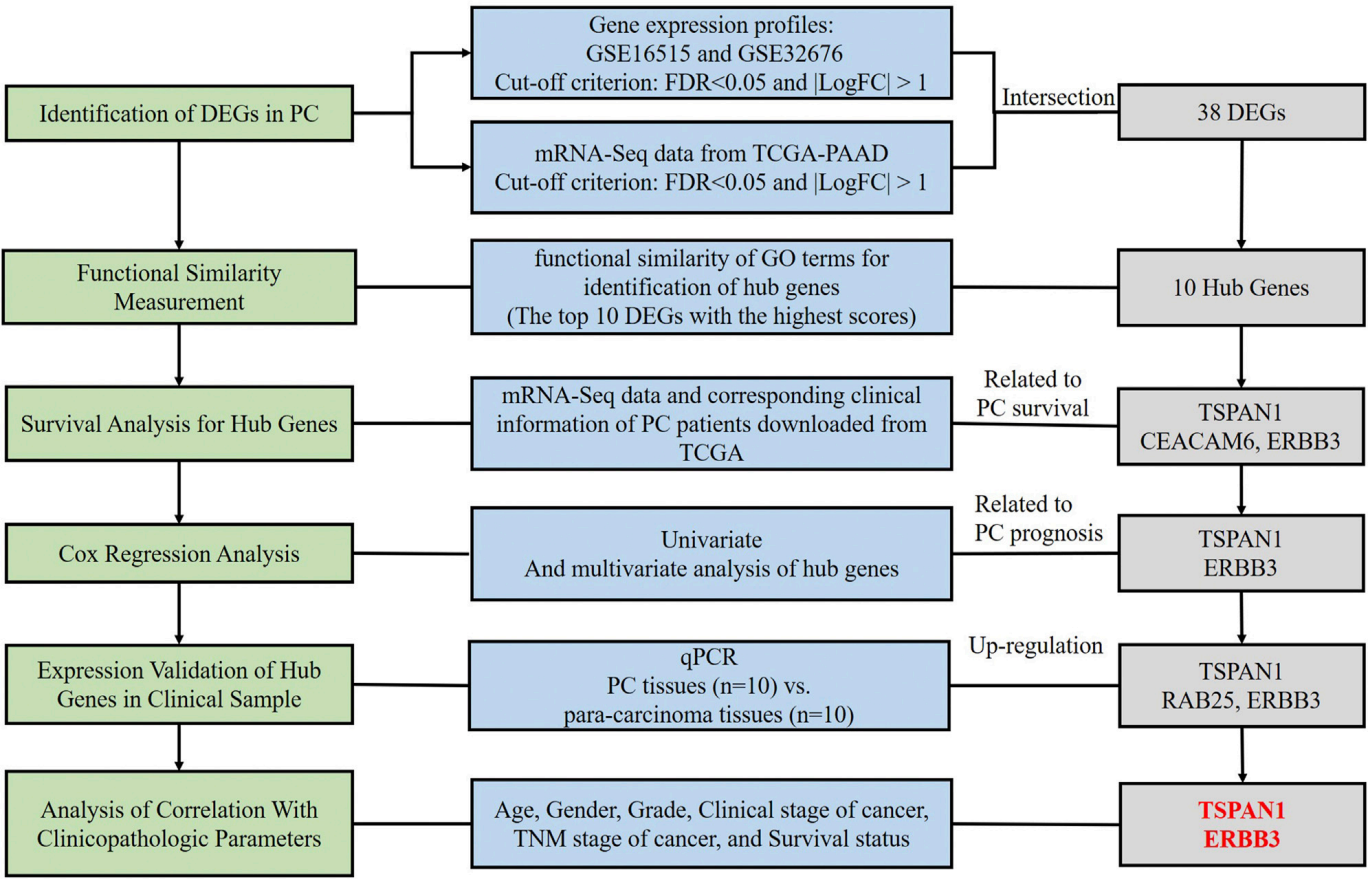
Flow chart of the study.

In the present study, we identified differentially expressed genes (DEGs) in PC based on a comprehensive analysis of Gene Expression Omnibus (GEO) and The Cancer Genome Atlas Pancreatic Cancer (TCGA-PAAD), and obtained the hub genes through measurement of functional similarity. Subsequently, survival analysis and Cox regression analysis were applied for the identification of prognosis-related hub genes. Statistics analysis in different subgroups and qPCR detection in clinical samples were used to verify the correlation between hub genes and clinical parameters. Therefore, this study was designed to explore novel potential prognosis predictors using bioinformatics and validation in clinical samples (Figure 1).

## Materials and Methods

### Data Extraction and Analysis of GEO Datasets

PC and adjacent non-tumor tissue gene expression profiles of GSE16515 [5] and GSE32676 [6] were obtained from the GEO database. The DEGs between PC tissues and adjacent non-tumor tissues from the two cohort profile data sets (GSE16515 and GSE32676) were screened using the limma package in R [7]. The corresponding clinical information is as follows in Supplementary Tables S1, S2.

### Validation in TCGA Datasets

The PC gene expression profile and corresponding clinical data for pancreatic cancer were collected from Firebrowse (http://firebrowse.org). The demographic and clinical characteristics of the corresponding PC patients are as follows in Supplementary Table S3. The RNA-Seq by Expectation-Maximization (RSEM) expression values were used for statistical analysis. The DEGs between PC tissues and adjacent non-tumor tissues from TCGA datasets were identified using the limma package. Heatmaps of common DEGs among the TCGA-PAAD datasets and 2 GEO datasets were developed by pheatmap package [8].

### Identification of Hub Genes Based on the Semantic Similarities of Gene Ontology Terms

After integrated analysis, we performed the measurement of the functional similarity among the DEGs in common to identify the hub genes using the GOSemSim package, which was based on the semantic similarities of Gene Ontology (GO) terms used for gene annotation [9]. The top ten DEGs with the higher average functional similarity were considered as the hub genes.

### Functional Analysis of Hub Genes

We performed Kaplan–Meier curves to compare overall survival between the high and low expression group for hub genes using the survival package in R [10], and P values were calculated by the log-rank test. The volcano plots were generated to visualize expression differences for discrete variables by the ggplot2 package in R [11]. Univariate Cox regression analysis was applied to assess the effect of hub gene expression and clinical-pathological factors on survival rates using the survival package. Multivariate Cox analysis was used to analyze the effects of hub gene expression on survival and other clinical features (gender, age, grade, TNM stage, clinical stage, vital status, etc.).

### Validation of the Hub Genes by Quantitative Real-Time RT-PCR (qRT-PCR)

A total of 10 PC patients were recruited for tumor and adjacent non-tumor tissue collection from the China-Japan Union Hospital of Jilin University, Changchun, China. This study was approved by the Ethics Committee of China-Japan Union Hospital of Jilin University (2021-KYLL-030009), and each patient consented to a written informed consent form. The clinicopathological characteristics of 10 PC patients were shown in Supplementary Table S4. High-quality total RNA from PC tissues was extracted using Trizol (Invitrogen, United States), and was measured using the NanoDrop 2000 spectrophotometer (Thermo Scientific, United States). Total RNA was reverse transcribed to cDNA with First Strand cDNA Synthesis Kit (Sangon, China), and qRT-PCR was performed using 2 x SG Fast qPCR Master Mix (Sangon, China) and Roche LightCycler 480 System (Roche, United States). Relative expression of the target gene was assessed by fold change using relative gene expression algorithms (fold change = 2^−ΔΔCt^, ΔCt = Ct_target_-Ct_GAPDH_, ΔΔCt = ΔCt_tumor_-ΔCt_non-tumor_). All primers for qRT-PCR were listed in Supplementary Table S5.

### Statistical Analysis

Statistical analysis of the results and boxplots was performed using GraphPad Prism 7.0 software. The comparisons of the mean values of the analyzed parameters were performed using two-tailed Student’s t-test and one-way ANOVA. The false discovery rate (FDR) controlling was used to correct the p-value with the Benjamini Hochberg algorithm implemented in R 4.0.3 suite (Lucent Technologies). FDR<0.05, P-value<0.05, and |logFC| (fold change) > 1 was considered statistically significant.

## Results

### Screening and Verification of Differentially Expressed Genes in GEO Datasets and TCGA-PAAD Datasets

**FIGURE 2:**
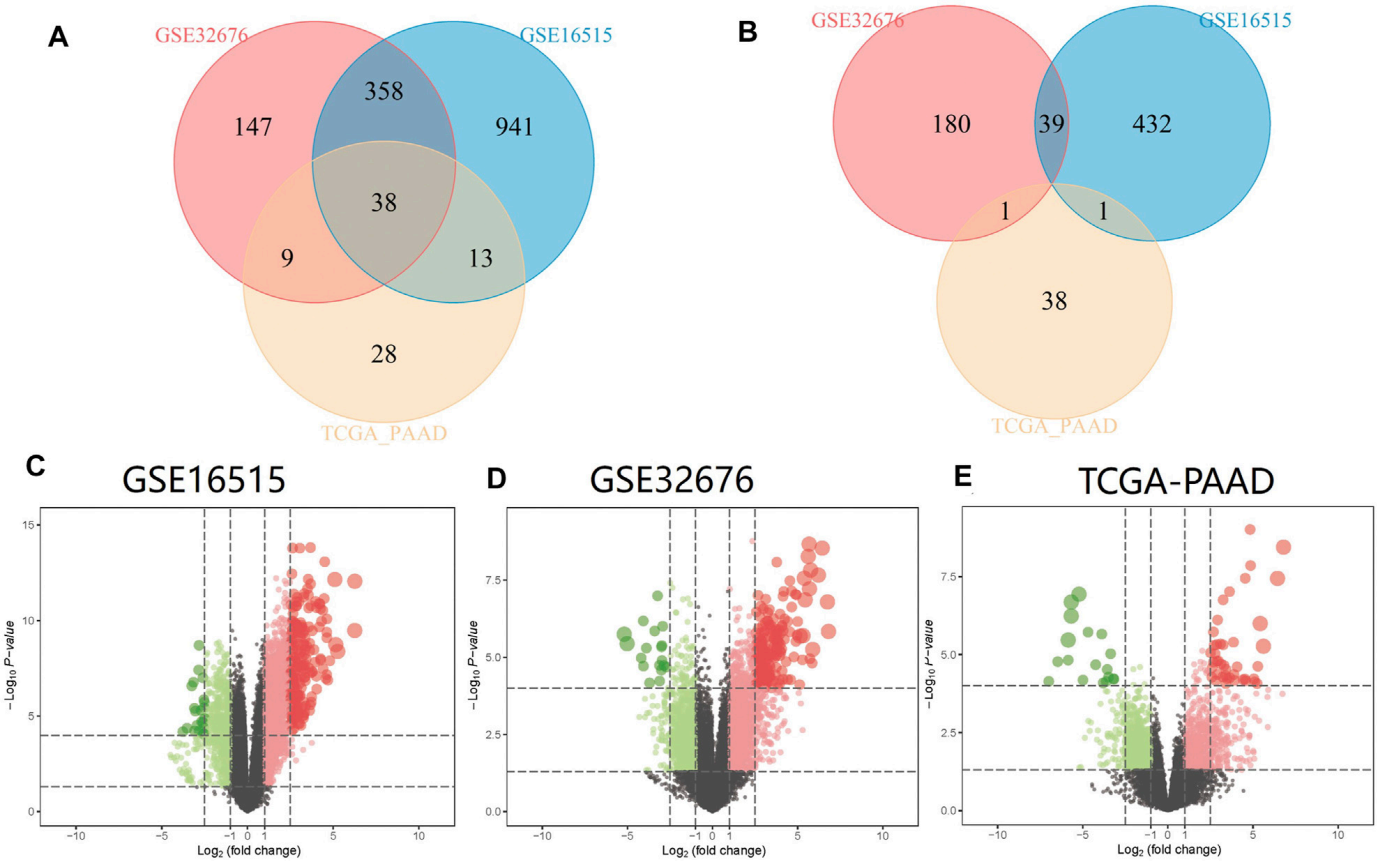
Identification of common DEGs in mRNA expression profiling datasets GSE16515, GSE32676, and TCGA-PAAD. Volcano plots and Venn diagrams of differentially expressed genes. **(A)** Venn diagram of upregulated DEGs between GSE16515, GSE32676, and TCGA-PAAD datasets; **(B)** Venn diagram of downregulated DEGs between GSE16515, GSE32676, and TCGA-PAAD datasets; **(C)** Volcano plot of DEGs of GSE16515 datasets; **(D)** Volcano plot of DEGs of GSE32676 datasets; **(E)** Volcano plot of DEGs of TCGA-PAAD datasets. Upregulated genes are marked in red; downregulated genes are marked in light green.

**FIGURE 3:**
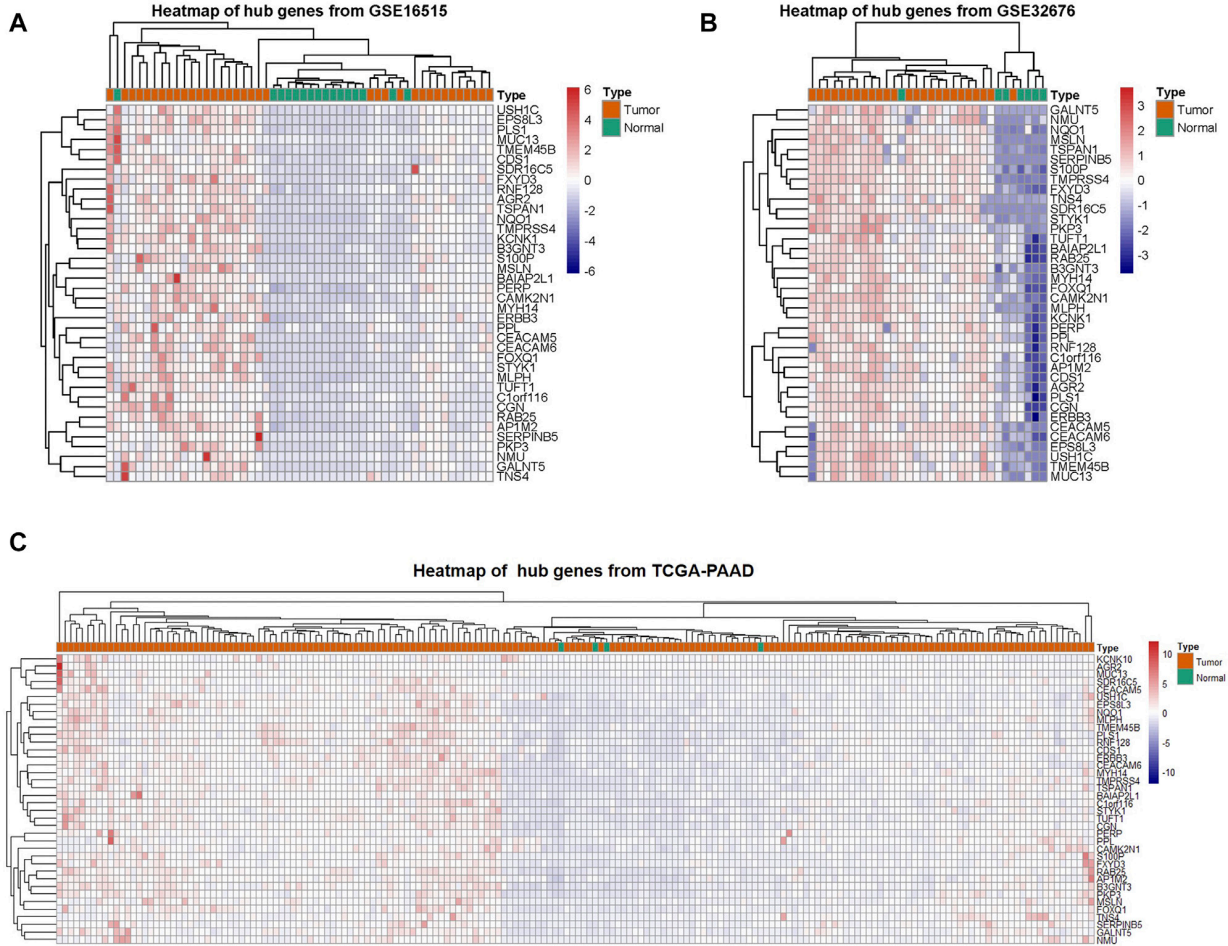
Clustering of the 38 DEGs in PAAD tissues vs. adjacent non-tumor tissues across each independent dataset. **(A–C)** A hierarchical clustering heat map showing the DEGs in common in PC tissues compared to adjacent non-tumor tissues.

PC and adjacent non-tumor tissue gene expression profiles of GSE16515 and GSE32676 were obtained from NCBI-GEO. We used *p* < 0.05, FDR<0.05 and |logFC|>1 as cut-off criterion. After integrated bioinformatics analysis, a total of 38 up-regulated DEGs was identified from the two GEO profile datasets and TCGA-PAAD RNA-Seq expression datasets (Table 1; Figure 2). We developed heatmaps of the 38 up-regulated DEGs in common based on the expression profiles, showing the significantly differential distribution of the 38 DEGs (Figure 3).

**TABLE 1:** The Co-upregulated DEGs in mRNA expression profiling datasets GSE16515, GSE32676, and TCGA-PAAD.

	Gene symbol
Co-upregulated DEGs	S100P, CEACAM5, CEACAM6, TMPRSS4, SERPINB5, MSLN, SDR16C5, AGR2, TSPAN1, NQO1, NMU, FXYD3, EPS8L3, GALNT5, TMEM45B, MLPH, MUC13, FOXQ1, STYK1, KCNK1, CAMK2N1, BAIAP2L1, RAB25, PLS1, C1orf116, B3GNT3, PKP3, CGN, USH1C, TUFT1, PERP, TNS4, ERBB3, CDS1, PPL, MYH14, RNF128, AP1M2

### Identification of Hub Genes Based on the Semantic Similarities of Gene Ontology Terms

**FIGURE 4:**
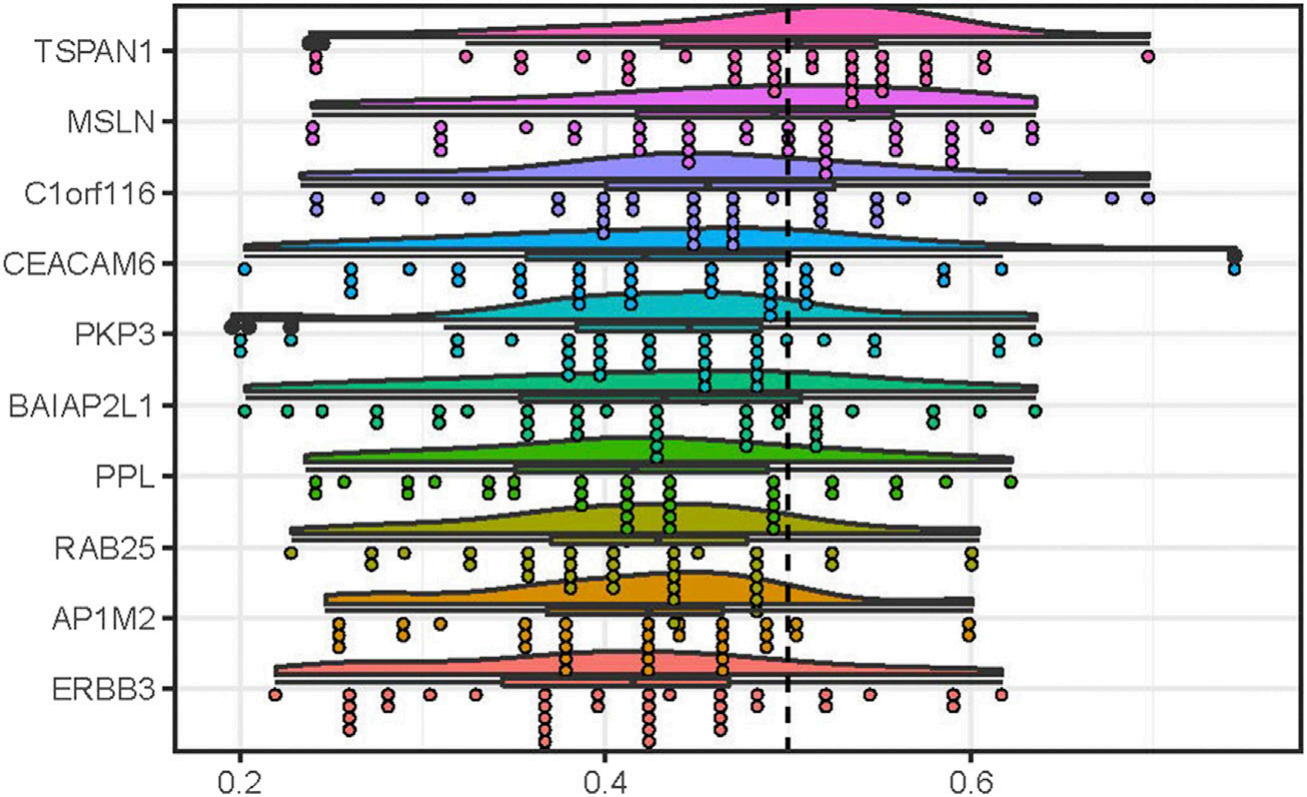
Summary of functional similarities of the DEGs in common in PC. The rainclouds were performed to reflect the distributions of functional similarities of 10 genes with top 10 scores, which were considered as the hub genes in PC. The dashed line represents the cutoff value.

We performed the measurement of the functional similarity among the common DEGs to identify the hub genes using the GOSemSim package. The results showed that 10 DEGs including TSPAN1, MSLN, C1orf116, PKP3, CEACAM6, BAIAP2L1, PPL, RAB25, ERBB3, and AP1M2, had the higher average functional similarity, which was considered as the hub genes (Figure 4). The distributions of functional similarities were summarized as Supplementary Table S7.

### TSPAN1 and ERBB3 Acts as Independent Prognostic Factors for Poor Survival of Pancreatic Cancer

**FIGURE 5:**
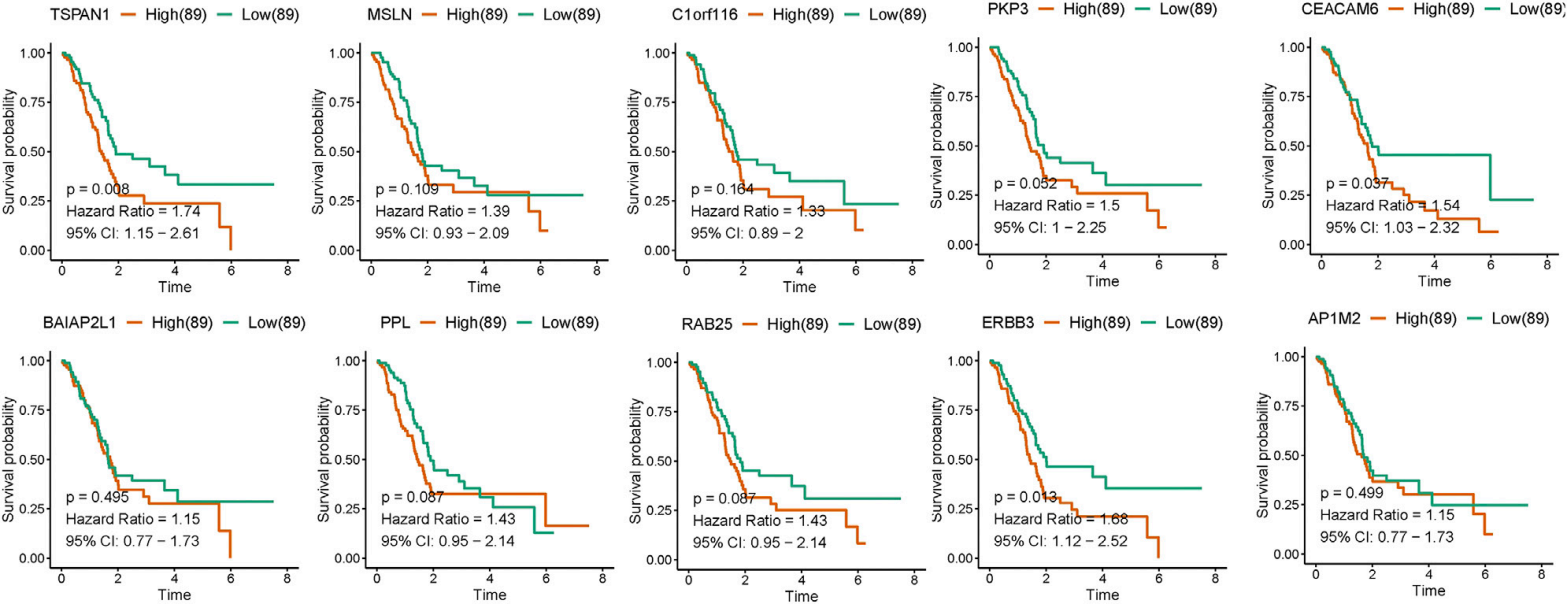
Kaplan–Meier curves for the survival of PAAD patients according to hub gene expression based on TCGA-PAAD datasets. Patients were divided into high and low hub gene expression groups using the median value of hub gene expression as the cutoff. Survival analysis and subgroup analysis according to grade, and TNM stage were performed based on Kaplan–Meier curves.

Survival analysis results showed that high-level expression of TSPAN1 (*p* = 0.008), CEACAM6 (*p* = 0.037), and ERBB3 (*p* = 0.013) was all associated with poor overall survival of PC patients (Figure 5 and Supplementary Table S6). Moreover, a further subgroup analysis showed that high TSPAN1 expression was associated with poor overall survival of patients with G1 tumors (*p* = 0.035), and N1 stage (*p* = 0.027); high ERBB3 expression was associated with poor overall survival of patients with N1 stage (*p* = 0.011); high CEACAM6 expression was associated with poor overall survival of patients with G1 tumors (*p* = 0.002) (Supplementary Figure 1). Univariate analysis results showed that some clinical characteristics including T stage (*p* = 0.026), N stage (*p* = 0.005), TSPAN1 (*p* = 0.008), CEACAM6 (*p* = 0.039), as well as ERBB3 (*p* = 0.014) expression were associated with poor overall survival (Table 2). Subsequently, clinical characteristics with a P-value < 0.05 were included for multivariate analysis, and the results showed that TSPAN1 (*p* = 0.04), ERBB3 (*p* = 0.046), and N stage (*p* = 0.012) expression were associated with poor overall survival in PC (Table 2).

**TABLE 2:** Univariate and multivariate analyses of clinicopathological characteristics, hub genes with overall survival in TCGA PAAD cohort.

Parameters	Univariate analysis		Multivariate analysis	
TCGA PAAD set	HR (95% CI)	P-value	HR (95% CI)	P-value
TSPAN1	1.485 (1.106–1.992)	**0.008**	1.364 (1.014–1.835)	**0.04**
MSLN	1.267 (0.948–1.694)	0.11		
C1orf116	1.228 (0.919–1.642)	0.166		
PKP3	1.332 (0.995–1.784)	0.054		
CEACAM6	1.363 (1.016–1.827)	**0.039**	1.104 (0.817–1.49)	0.52
BAIAP2L1	1.106 (0.828–1.478)	0.494		
PPL	1.288 (0.962–1.725)	0.089		
RAB25	1.287 (0.962–1.722)	0.089		
ERBB3	1.446 (1.078–1.938)	**0.014**	1.35 (1.005–1.814)	**0.046**
AP1M2	1.105 (0.827–1.475)	0.499		
Gender	0.872 (0.653–1.163)	0.35		
Age	1.208 (0.904–1.614)	0.202		
Histologic grade	1.343 (0.988–1.824)	0.06		
Pathologic T stage	1.663 (1.062–2.604)	**0.026**	1.147 (0.724–1.816)	0.560
Pathologic N Stage	1.698 (1.177–2.449)	**0.005**	1.647 (1.16–2.431)	**0.012**
Pathologic M stage	1.035 (0.377–2.846)	0.947		
Tumor stage	0.805 (0.356–1.822)	0.603		

Bold values of *p* ≤ 0.05 indicate statistically significant correlations.

### Validation of Hub Gene Expression in Clinical Samples

**FIGURE 6:**
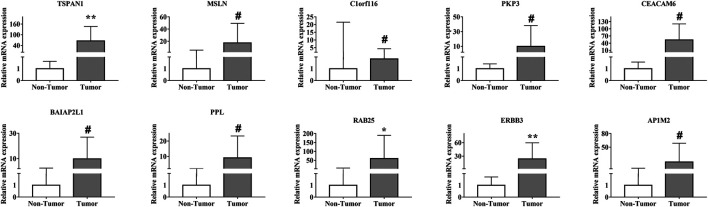
Validation of the expression of the 10 hub genes in 10 paired PC and para-carcinoma tissues by qPCR. All samples were normalized to the expression of GAPDH. The relative expression level of each gene was analyzed using the 2^−ΔΔCt^ method. *p < 0.05, **p < 0.01, ^#^ *p* > 0.05.

To validate the bioinformatics results, we analyzed the expression of the 10 hub genes in carcinoma and para-carcinoma tissues by qPCR. The results showed that the expression levels of TSPAN1 (*p* < 0.01), RAB25 (*p* < 0.05), and ERBB3 (*p* < 0.01) were significantly upregulated in the PC tissues (Figure 6), which is consistent with the bioinformatics analysis results.

### Analysis of Correlation Between TSPAN1, ERBB3 Expression, and Clinicopathologic Parameters

**FIGURE 7:**
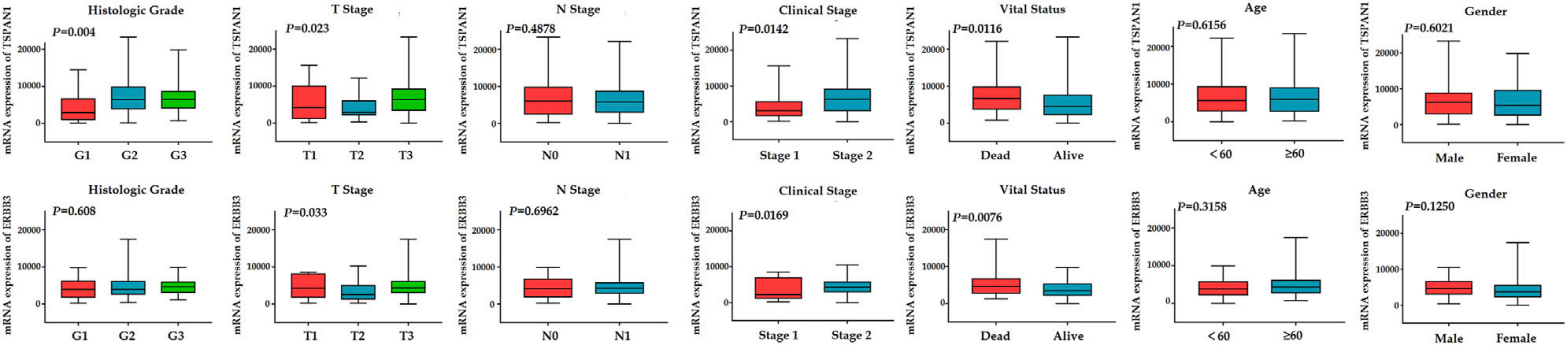
Boxplots for differential expression of TSPAN1 and ERBB3 according to age, gender, grade, clinical stage of cancer, TNM stage of cancer, and survival status based on TCGA-PAAD datasets. P-values were calculated using a two-tailed Student’s t-test or one-way ANOVA.

To further evaluate the association between the expression levels of TSPAN1, ERBB3 expression, and clinicopathologic parameters of PC including patient age, gender, grade, clinical stage of cancer (stage 1 and stage 2), TNM stage of cancer, and survival status, we drew boxplots of the hub gene expression based on the TCGA–PAAD datasets (Figure 7). The results revealed that TSPAN1 expression was significantly associated with histologic grade (*p* = 0.004), T stage (*p* = 0.023), clinical stage (*p* = 0.0142), and vital status (*p* = 0.0116) by two-tailed Student’s t-test or one-way ANOVA; ERBB3 expression correlated significantly with T stage (*p* = 0.033), clinical stage (*p* = 0.0169), and vital status (*p* = 0.0076) by two-tailed Student’s t-test or one-way ANOVA.

## Discussion

In recent years, the continuous expansion of public databases including TCGA and GEO databases, has provided new means for tumor research and valuable first-hand data for clinicians' evidence-based practice and clinical research. In the study, we identified 38 DEGs in common in PC, which were all up-regulated after integrated analysis of GEO and TCGA-PAAD datasets. Through assessment of functional similarity for common DEGs, we found that the top ten DEGs had the highest average functional similarity, and were considered as the hub genes of 38 DEGs. Then, survival analysis, Cox univariate and multivariate analysis results indicated that the expression of TSPAN1 and ERBB3 was both negatively correlated with the prognosis of pancreatic cancer, especially those with N1 and/or G1. In addition, the expression level of TSPAN1 and ERBB3 was both correlated with patients’ survival status, T stage, clinical stage, and TSPAN1 was correlated with histologic grade. Detection results in clinical samples by qPCR also demonstrated that TSPAN1 and ERBB3 were upregulated in PC tissues. Therefore, TSPAN1 and ERBB3 could be involved in the prognosis of PC.

Tetraspanins expressed on the cell surface, are 4-transmembrane spanning proteins with a relative molecular mass of 25 × 10^3^–50×10^3^, which are widely expressed in different species including at least 33 different family members of mammals [12, 13]. They interact within the molecule or with other proteins to regulate various basic cellular physiological processes, such as cell differentiation, cell migration, and signal transduction. TSPAN1 (Tetraspanin 1) is a member of the Tetraspanins family of proteins whose important feature is their ability to aggregate with one another or various other transmembrane receptors, to become TSPAN-enriched microdomains (TEMs), which are essential in determining the fundamental biological activities such as cell adhesion, proliferation, and cell motility [12, 14].

A few studies have reported that TSPAN1 could play a significant role in the progression of PC [15]. A study by Hou et al. showed that transfection with siRNA-targeting TSPAN1 significantly decreased proliferation, increased apoptosis, and reduced migration and invasion of AsPC-1 and PANC-1 cells, which suggested that TSPAN1 was involved in the PC of migration and invasion [16]. Studies performed by Tian J et al. and Zhang X et al. also reached a similar conclusion in Capan2, BxPC3, and SW1990 cells through silencing TSPAN1 [17, 18]. Another study has identified TSPAN1 as one of the potential diagnostic markers and was expressed at a high level in PC tissues and cells, which was consistent with our study results [19]. Ma C et al. identified TSPAN1 could be involved in PC progression and act as a critical biomarker for diagnosing and predicting patient survival with PC through weighted gene co-expression network analysis [20]. Even so, the role of TSPAN1 in the prognosis of PC is not yet clear. In the present study, we found that the expression levels of TSPAN1 were significantly upregulated in PC based on TCGA-PAAD and GEO database, and the result was validated in clinical samples. Moreover, PC patients with high TSPAN1 expression had significantly lower survival than those with low TSPAN1 expression. The progression of PC, especially histologic grade, pathology T stage clinical stage, and vital status were closely related to TSPAN1 expression, which provides for TSPAN1 as a valuable surveillance indicator for prognosis of PC.

ERBB3 (Erb-b2 receptor tyrosine kinase 3), as a member of the ErbB family, is highly expressed in a variety of common tumors and can induce resistance to a variety of tumor therapeutic drugs when activated [21, 22]. In the previous study, the inhibition of ERBB3 by miR-148a was reported to repress the phosphorylation of ERK1/2 and AKT, thereby inhibiting the proliferation and migration of PC [23]. *In vivo* and *in vitro* experiments from another study conducted by Liles JS confirmed that high expression of ERBB3 promotes tumorigenesis of pancreatic adenocarcinoma [24]. We found that ERBB3 was highly expressed in PC tissues and negatively correlative with an overall survival rate of PC patients in our study. Moreover, the expression level of ERBB3 was involved in the prognosis and progression of PC including pathology T stage, clinical stage, and vital status.

## Conclusion

In conclusion, through the integrated analysis of the TCGA-PAAD and the GEO datasets, as well as the validation in clinical samples, high TSPAN1, and ERBB3 expression could act as independent prognostic factors for poor overall survival in PC. In the future, we will explore the potential mechanism of TSPAN1 and ERBB3 in PC by further validation.

## Data Availability

The original contributions presented in the study are included in the article/Supplementary Material, further inquiries can be directed to the corresponding author.
