# Supplementary material for: Screening and Validation of Independent Predictors of Poor Survival in Pancreatic Cancer
Source: Pathol Oncol Res. 2021 Jul 12;27:1609868. doi: 10.3389/pore.2021.1609868 (PMC8310909; doi:10.3389/pore.2021.1609868)
Supplement: Supplementary file 1 [file DataSheet2.docx]

**Supplementary Figure 1 Kaplan–Meier curves for survival of PAAD patients according to hub gene expression based on TCGA-PAAD datasets.**

(A)TSPAN1 subgroup survival analysis; (B)CEACAM6 subgroup survival analysis; (C)ERBB3 subgroup survival analysis; Patients were divided into high and low hub gene expression groups using the median value of hub gene expression as the cutoff. Survival analysis and subgroup analysis according to grade, and TNM stage were performed based on Kaplan–Meier curves.


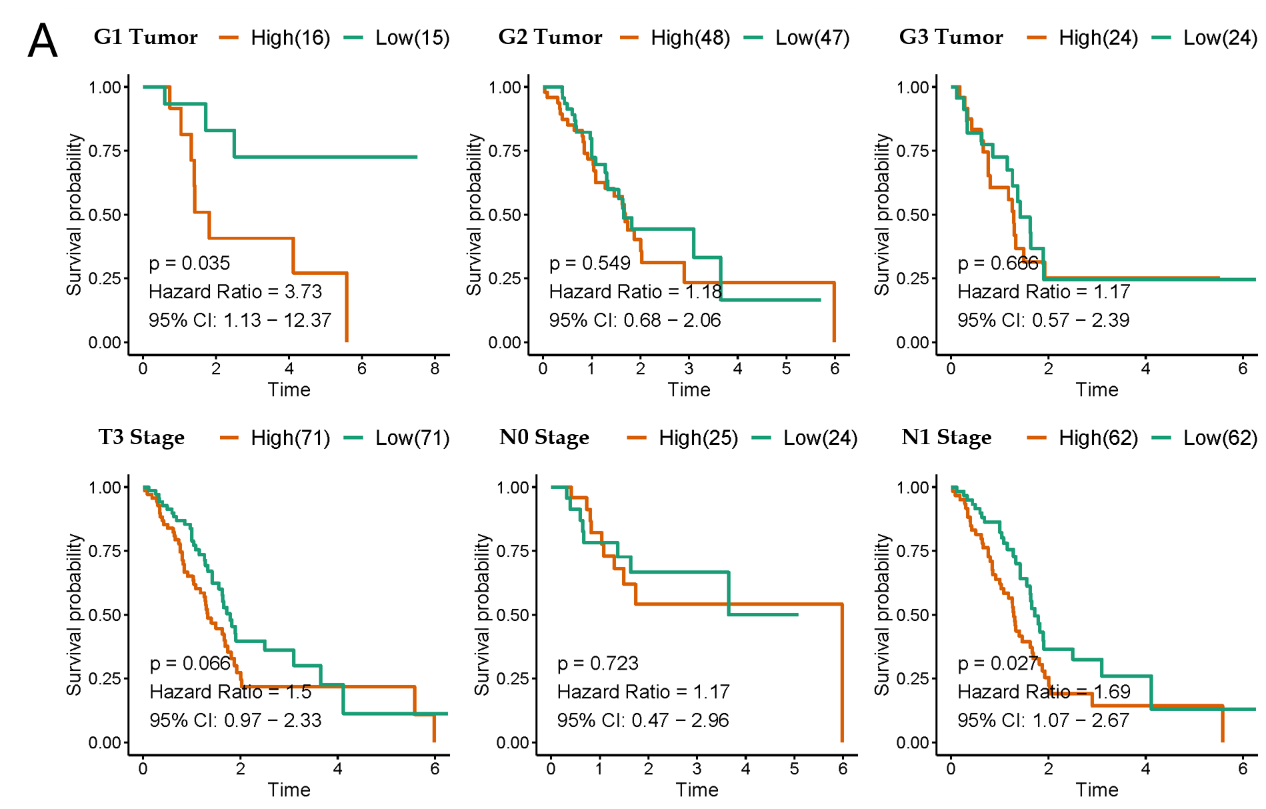


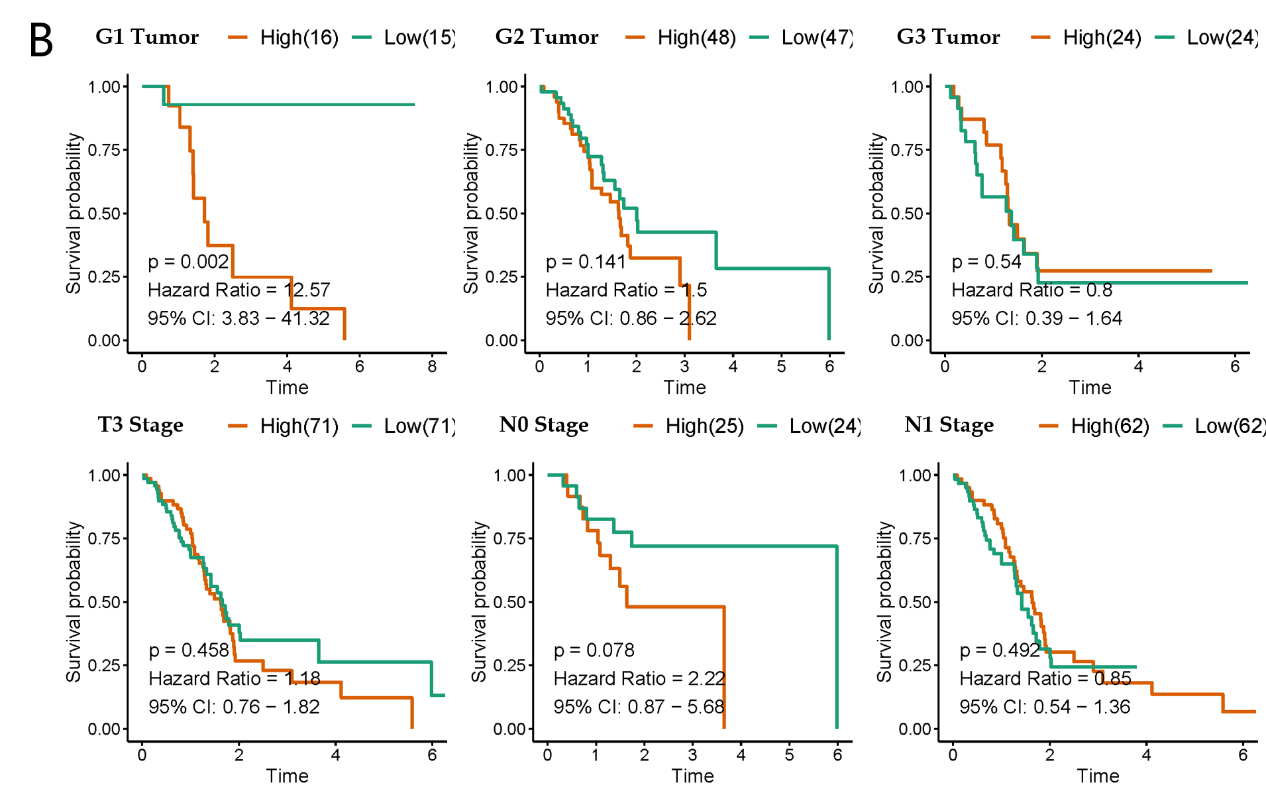


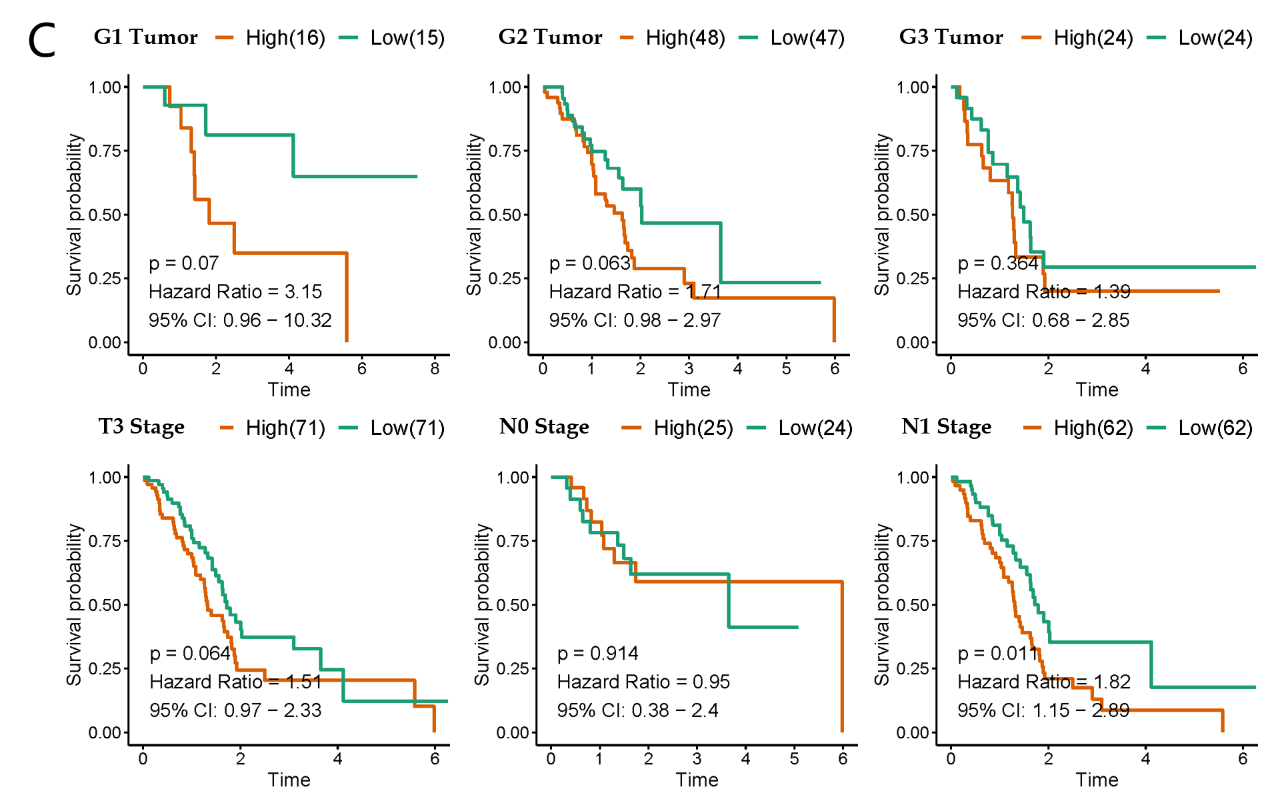


**Supplementary Table 1 Clinicopathological characteristics of PC samples from GES16515**

| Accession | Tissue | Sex | Age |
| --- | --- | --- | --- |
| GSM414934 | Normal Tissue in Pancreatic Cancer Sample | Male | 74 |
| GSM414933 | Tumor Tissue in Pancreatic Cancer Sample | Male | 74 |
| GSM414935 | Tumor Tissue in Pancreatic Cancer Sample | Male | 64 |
| GSM414936 | Tumor Tissue in Pancreatic Cancer Sample | Female | 66 |
| GSM414942 | Normal Tissue in Pancreatic Cancer Sample | Male | 84 |
| GSM414941 | Tumor Tissue in Pancreatic Cancer Sample | Male | 84 |
| GSM414940 | Normal Tissue in Pancreatic Cancer Sample | Male | 71 |
| GSM414939 | Tumor Tissue in Pancreatic Cancer Sample | Male | 71 |
| GSM414928 | Normal Tissue in Pancreatic Cancer Sample | Male | 51 |
| GSM414927 | Tumor Tissue in Pancreatic Cancer Sample | Male | 51 |
| GSM414943 | Tumor Tissue in Pancreatic Cancer Sample | Female | 76 |
| GSM414948 | Tumor Tissue in Pancreatic Cancer Sample | Female | 57 |
| GSM414926 | Tumor Tissue in Pancreatic Cancer Sample | Male | 74 |
| GSM414944 | Tumor Tissue in Pancreatic Cancer Sample | Male | 81 |
| GSM414949 | Tumor Tissue in Pancreatic Cancer Sample | Male | 58 |
| GSM414950 | Tumor Tissue in Pancreatic Cancer Sample | Male | 58 |
| GSM414951 | Tumor Tissue in Pancreatic Cancer Sample | Female | 72 |
| GSM414953 | Normal Tissue in Pancreatic Cancer Sample | Male | 73 |
| GSM414952 | Tumor Tissue in Pancreatic Cancer Sample | Male | 73 |
| GSM414955 | Normal Tissue in Pancreatic Cancer Sample | Female | 79 |
| GSM414954 | Tumor Tissue in Pancreatic Cancer Sample | Female | 79 |
| GSM414958 | Tumor Tissue in Pancreatic Cancer Sample | Male | 49 |
| GSM414967 | Tumor Tissue in Pancreatic Cancer Sample | Female | 69 |
| GSM414959 | Tumor Tissue in Pancreatic Cancer Sample | Female | 74 |
| GSM414961 | Tumor Tissue in Pancreatic Cancer Sample | Female | 71 |
| GSM414957 | Normal Tissue in Pancreatic Cancer Sample | Female | 58 |
| GSM414956 | Tumor Tissue in Pancreatic Cancer Sample | Female | 58 |
| GSM414963 | Normal Tissue in Pancreatic Cancer Sample | Male | 75 |
| GSM414962 | Tumor Tissue in Pancreatic Cancer Sample | Male | 75 |
| GSM414960 | Tumor Tissue in Pancreatic Cancer Sample | Male | 61 |
| GSM414966 | Normal Tissue in Pancreatic Cancer Sample | Male | 68 |
| GSM414965 | Tumor Tissue in Pancreatic Cancer Sample | Male | 68 |
| GSM414968 | Tumor Tissue in Pancreatic Cancer Sample | Female | 67 |
| GSM414970 | Normal Tissue in Pancreatic Cancer Sample | Male | 55 |
| GSM414969 | Tumor Tissue in Pancreatic Cancer Sample | Male | 55 |
| GSM414972 | Normal Tissue in Pancreatic Cancer Sample | Female | 74 |
| GSM414971 | Tumor Tissue in Pancreatic Cancer Sample | Female | 74 |
| GSM414973 | Tumor Tissue in Pancreatic Cancer Sample | Female | 79 |
| GSM414975 | Normal Tissue in Pancreatic Cancer Sample | Male | 63 |
| GSM414974 | Tumor Tissue in Pancreatic Cancer Sample | Male | 63 |
| GSM414947 | Normal Tissue in Pancreatic Cancer Sample | Male | 73 |
| GSM414946 | Tumor Tissue in Pancreatic Cancer Sample | Male | 73 |
| GSM414930 | Normal Tissue in Pancreatic Cancer Sample | Male | 54 |
| GSM414929 | Tumor Tissue in Pancreatic Cancer Sample | Male | 54 |
| GSM414938 | Normal Tissue in Pancreatic Cancer Sample | Female | 59 |
| GSM414937 | Tumor Tissue in Pancreatic Cancer Sample | Female | 59 |
| GSM414945 | Tumor Tissue in Pancreatic Cancer Sample | Male | 60 |
| GSM414924 | Tumor Tissue in Pancreatic Cancer Sample | Female | 57 |
| GSM414925 | Tumor Tissue in Pancreatic Cancer Sample | Male | 69 |
| GSM414932 | Normal Tissue in Pancreatic Cancer Sample | Male | 63 |
| GSM414931 | Tumor Tissue in Pancreatic Cancer Sample | Male | 63 |
| GSM414964 | Tumor Tissue in Pancreatic Cancer Sample | Male | 72 |

**Supplementary Table 2 Clinicopathological characteristics of PC samples from GSE32676**

| Accession | Source name | Ajcc stage | Grade | Tumor content |
| --- | --- | --- | --- | --- |
| GSM811004 | human pancreatic tumor | 2B | 2 | 90 |
| GSM811005 | human pancreatic tumor | 1B | 2 | 90 |
| GSM811006 | human pancreatic tumor | 2A | 1 | 80 |
| GSM811007 | human pancreatic tumor | 2B | 2 | 80 |
| GSM811008 | human pancreatic tumor | 2B | 2 | 80 |
| GSM811009 | human pancreatic tumor | 2B | 3 | 80 |
| GSM811010 | human pancreatic tumor | 1B | 2 | 75 |
| GSM811011 | human pancreatic tumor | 2B | 3 | 75 |
| GSM811012 | human pancreatic tumor | 2B | 3 | 75 |
| GSM811013 | human pancreatic tumor | 2B | 2 | 70 |
| GSM811014 | human pancreatic tumor | 2B | 2 | 60 |
| GSM811015 | human pancreatic tumor | 2A | 2 | 60 |
| GSM811016 | human pancreatic tumor | 2B | 3 | 60 |
| GSM811017 | human pancreatic tumor | 2A | 2 | 60 |
| GSM811018 | human pancreatic tumor | 2A | 3 | 50 |
| GSM811019 | human pancreatic tumor | 2B | 2 | 50 |
| GSM811020 | human pancreatic tumor | 2B | 2 | 50 |
| GSM811021 | human pancreatic tumor | 2B | 3 | 50 |
| GSM811022 | human pancreatic tumor | 2A | 2 | 45 |
| GSM811023 | human pancreatic tumor | 2B | 2 | 40 |
| GSM811024 | human pancreatic tumor | 2B | 2 | 40 |
| GSM811025 | human pancreatic tumor | 2B | 3 | 35 |
| GSM811026 | human pancreatic tumor | 2B | 2 | 35 |
| GSM811027 | human pancreatic tumor | 2B | 2 | 35 |
| GSM811028 | human pancreatic tumor | 2A | 2 | 35 |
| GSM811029 | non-malignant pancreas | NA | NA | NA |
| GSM811030 | non-malignant pancreas | NA | NA | NA |
| GSM811031 | non-malignant pancreas | NA | NA | NA |
| GSM811032 | non-malignant pancreas | NA | NA | NA |
| GSM811033 | non-malignant pancreas | NA | NA | NA |
| GSM811034 | non-malignant pancreas | NA | NA | NA |
| GSM811035 | non-malignant pancreas | NA | NA | NA |

**Supplementary Table 3 Demographic and clinical characteristics of TCGA-PAAD cohort**

| Characteristics | | Number of patients (%) |  |
| --- | --- | --- | --- |
| Age | ≤55 | 38(21.35) |  |
|  | >55 | 140(78.65) |  |
| Gender | Female | 80(44.94) |  |
|  | Male | 98(55.06) |  |
| Vital status | Alive | 85(47.75) |  |
|  | Dead | 93(52.25) |  |
| Pathology T stage | T1 | 7(3.93) |  |
|  | T2 | 24(13.48) |  |
|  | T3 | 142(79.78) |  |
|  | T4 | 3(1.69) |  |
|  | Tx | 1(0.56) |  |
|  | NA | 1(0.56) |  |
| Pathology N stage | N0 | 49(27.53) |  |
|  | N1 | 124(69.66) |  |
|  | Nx | 4(2.25) |  |
|  | NA | 1(0.56) |  |
| Pathology M stage | M0 | 80(44.94) |  |
|  | M1 | 4(2.25) |  |
|  | Mx | 94(52.81) |  |
| Stage | I | 21(11.80) |  |
|  | II | 147(82.58) |  |
|  | III | 4(2.25) |  |
|  | IV | 4(2.25) |  |
|  | NA | 2(1.12) |  |
| Grade | G1 | 31(17.42) |  |
|  | G2 | 95(53.37) |  |
|  | G3 | 48(26.97) |  |
|  | G4 | 2(1.12) |  |
|  | Gx | 2(1.12) |  |

**Supplementary Table 4** **Clinicopathological characteristics of 10 PC patients**

| Case | Age | Gender | Pathological Diagnosis  (Differentiation Status) | Tumor Size(cm) | Vascular Invasion | Neural Invasion |
| --- | --- | --- | --- | --- | --- | --- |
|  |  |  |  |  |  |  |
| 1 | 45 | male | poorly differentiated | 5.8×5×4 | - | - |
| 2 | 37 | male | poorly differentiated | 10×7×5.5 | - | - |
| 3 | 52 | male | Moderately differentiated | 8×6×6 | + | - |
| 4 | 43 | male | moderately differentiated | 3×3×2.8 | - | - |
| 5 | 52 | female | moderately/poorly differentiated | ? | - | - |
| 6 | 62 | female | moderately differentiated | 4.5×4×3.5 | - | - |
| 7 | 43 | male | moderately/poorly differentiated | 9×8×6 | - | - |
| 8 | 53 | male | moderately differentiated | 5.5×5×3 | - | - |
| 9 | 65 | female | moderately differentiated | 6×5×5cm | - | - |
| 10 | 65 | male | poorly differentiated | ? | + | - |

Note: “?” represented for lack of relevant results; “+” represented positive results; “-” represented negative results.

**Supplementary Table 5 Primer sequences used for qRT-PCR amplification.**

| Primer | 5' > 3' |
| --- | --- |
| TSPAN1 | CATGCAGTTTGTCAACGTGGG |
|  | CACTTGCTCTCAGTCTTAGCAC |
| MSLNL | CCAACCCACCTAACATTTCCAG |
|  | CAGCAGGTCCAATGGGAGG |
| C1orf116 | CAGTCTCAACCAGGTACACAC |
|  | TAGTCGGCTGTTTGGGTCCT |
| PKP3 | GGCCGACTATGACACACTCTC |
|  | CTCGGGGAAACCTCAGTGG |
| CEACAM6 | TCAATGGGACGTTCCAGCAAT |
|  | CACTCCAATCGTGATGCCGA |
| BAIAP2L1 | AGAGCACCTACCGGAATGTTA |
|  | TGGCAATCTCACCGATCTTGG |
| PPL | CCGGAGCATCTCTAACAAGGA |
|  | GCATCCGCCTCTAGCACAT |
| RAB25 | AACCAAGCACCAGACCTATGC |
|  | CACTTTTGTTACCCACGAGCA |
| ERBB3 | GACCCAGGTCTACGATGGGAA |
|  | GTGAGCTGAGTCAAGCGGAG |
| AP1M2 | TGTCTTCATTCTGGACGTTAAGG |
|  | AGTGCTCAATCTTGCTCATGG |

**Supplementary Table 6 Expression data of hub genes and follow-up data of PC patients based on TCGA-PAAD datasets.**

| Sample ID | EVENT | OS | TSPAN1 | MSLN | C1orf116 | PKP3 | CEACAM6 | BAIAP2L1 | PPL | RAB25 | ERBB3 | AP1M2 |
| --- | --- | --- | --- | --- | --- | --- | --- | --- | --- | --- | --- | --- |
| TCGA-2J-AAB1-01A-11R-A41B-07 | Dead | 66 | 19011.7073 | 19610.2439 | 6810.732 | 4464.39 | 88335.61 | 3600.976 | 5979.512 | 3861.951 | 7187.805 | 3185.366 |
| TCGA-2J-AAB4-01A-12R-A41B-07 | Alive | 729 | 12214.6465 | 5010.101 | 2606.482 | 3392.256 | 42850.17 | 3702.862 | 3669.613 | 2855.64 | 3869.108 | 2347.222 |
| TCGA-2J-AAB6-01A-11R-A41B-07 | Dead | 293 | 9798.8304 | 12692.9825 | 4681.287 | 5234.503 | 3925.731 | 2330.994 | 4765.497 | 2511.111 | 1953.216 | 2011.696 |
| TCGA-2J-AAB8-01A-12R-A41B-07 | Alive | 80 | 5249.3537 | 6646.2985 | 2321.034 | 2508.108 | 55773.44 | 1871.68 | 2541.011 | 4705.993 | 6656.639 | 2556.052 |
| TCGA-2J-AAB9-01A-11R-A41B-07 | Dead | 627 | 1749.1961 | 2201.5005 | 1730.975 | 1788.853 | 21551.98 | 2546.624 | 3032.154 | 2126.474 | 2678.457 | 2082.53 |
| TCGA-2J-AABA-01A-21R-A41B-07 | Dead | 607 | 8184.7503 | 13126.0373 | 2008.455 | 2948.489 | 56363.24 | 2025.364 | 5338.344 | 2226.397 | 6947.863 | 1607.641 |
| TCGA-2J-AABE-01A-12R-A41B-07 | Alive | 676 | 10780.3299 | 26530.5997 | 3167.566 | 3578.233 | 29600.12 | 2743.333 | 3749.653 | 3853.862 | 5119.161 | 2769.231 |
| TCGA-2J-AABF-01A-31R-A41B-07 | Dead | 691 | 4182.8652 | 3551.3423 | 3716.842 | 2696.554 | 30820.94 | 2192.611 | 2671.746 | 2103.659 | 5710.286 | 1988.837 |
| TCGA-2J-AABH-01A-21R-A41B-07 | Alive | 1287 | 7571.8641 | 11449.9129 | 4462.544 | 4956.882 | 38893.73 | 4236.934 | 4994.774 | 5165.505 | 3081.011 | 3760.017 |
| TCGA-2J-AABI-01A-12R-A41B-07 | Alive | 969 | 1492.3803 | 29.7533 | 2111.393 | 4362.845 | 56.6038 | 3118.287 | 20733.67 | 2276.125 | 1108.491 | 727.5036 |
| TCGA-2J-AABK-01A-31R-A41B-07 | Alive | 484 | 9039.9828 | 5409.2863 | 3219.261 | 3476.784 | 29953.57 | 3738.177 | 2167.24 | 4878.762 | 4551.591 | 3065.778 |
| TCGA-2J-AABO-01A-21R-A41B-07 | Alive | 440 | 5805.2885 | 2419.8718 | 4088.141 | 7165.865 | 25591.35 | 2463.141 | 3397.436 | 2556.09 | 3288.462 | 1943.91 |
| TCGA-2J-AABP-01A-11R-A41B-07 | Alive | 463 | 14.7291 | 12.6249 | 5.2604 | 37.8748 | 217.7801 | 138.8743 | 17.3593 | 6.3125 | 18.9374 | 29.9842 |
| TCGA-2J-AABR-01A-11R-A41B-07 | Alive | 438 | 6003.7106 | 2977.7365 | 1076.685 | 1865.801 | 25459.49 | 2235.003 | 2881.262 | 1171.923 | 4443.414 | 1311.688 |
| TCGA-2J-AABT-01A-11R-A41B-07 | Alive | 319 | 2452.4406 | 6080.7259 | 1523.154 | 1349.187 | 10486.23 | 1747.184 | 1420.526 | 1429.912 | 3850.438 | 1011.89 |
| TCGA-2J-AABU-01A-11R-A41B-07 | Dead | 277 | 8594.9782 | 4179.0393 | 2018.559 | 2043.668 | 9665.939 | 3176.856 | 7990.175 | 2742.358 | 2175.764 | 1358.079 |
| TCGA-2J-AABV-01A-12R-A41B-07 | Dead | 652 | 1406.2992 | 615.748 | 1699.213 | 3045.669 | 2458.268 | 2927.559 | 1141.732 | 2866.142 | 3393.701 | 3234.646 |
| TCGA-2L-AAQA-01A-21R-A38C-07 | Dead | 143 | 6503.1168 | 12527.6493 | 5885.783 | 5106.777 | 60365.98 | 4573.497 | 3104.364 | 4796.3 | 4827.669 | 3276.895 |
| TCGA-2L-AAQE-01A-11R-A39D-07 | Dead | 684 | 12630.2132 | 17997.683 | 3056.997 | 6894.81 | 60175.63 | 3662.651 | 8504.634 | 4159.87 | 5637.164 | 3243.744 |
| TCGA-2L-AAQI-01A-12R-A39D-07 | Dead | 103 | 7261.058 | 7856.2267 | 4691.873 | 5215.621 | 66886.76 | 2442.378 | 5772.408 | 2896.164 | 7278.549 | 3000.136 |
| TCGA-2L-AAQJ-01A-12R-A39D-07 | Dead | 394 | 14094.0594 | 10619.802 | 4158.416 | 5048.515 | 93752.48 | 3902.475 | 2605.941 | 5811.881 | 8431.683 | 2783.168 |
| TCGA-2L-AAQL-01A-11R-A38C-07 | Dead | 292 | 7055.1289 | 33720.6313 | 3971.623 | 4909.547 | 62060.15 | 4392.719 | 3074.45 | 5923.799 | 5478.77 | 3146.897 |
| TCGA-2L-AAQM-01A-11R-A39D-07 | Alive | 1383 | 1988.1306 | 0 | 1817.211 | 974.4807 | 1090.801 | 932.9377 | 2348.961 | 852.2255 | 873.5905 | 2686.647 |
| TCGA-3A-A9I5-01A-11R-A38C-07 | Alive | 1794 | 325.9304 | 43.2173 | 1765.306 | 3266.507 | 4767.107 | 2691.477 | 1000.6 | 4185.474 | 5169.868 | 3226.891 |
| TCGA-3A-A9I7-01A-21R-A38C-07 | Alive | 1323 | 5435.7566 | 5091.716 | 2858.411 | 3662.722 | 8191.885 | 3703.956 | 2396.45 | 2225.275 | 4226.543 | 1611.581 |
| TCGA-3A-A9I9-01A-11R-A38C-07 | Dead | 634 | 9243.0595 | 12698.5836 | 2005.099 | 3097.45 | 17049.86 | 3814.731 | 3781.303 | 3037.394 | 5121.247 | 2639.094 |
| TCGA-3A-A9IB-01A-21R-A39D-07 | Dead | 224 | 8233.8003 | 13953.4415 | 1214.552 | 3371.964 | 8247.103 | 1791.394 | 3226.444 | 1413.282 | 4342.235 | 1242.366 |
| TCGA-3A-A9IC-01A-11R-A38C-07 | Dead | 738 | 14484.9383 | 8383.2099 | 2337.778 | 2409.877 | 13065.19 | 2720.494 | 2916.543 | 3477.037 | 3000.988 | 1289.877 |
| TCGA-3A-A9IH-01A-12R-A39D-07 | Alive | 1021 | 15699.1579 | 42198.0447 | 4729.455 | 5654.825 | 24302.39 | 2767.206 | 5712.129 | 3669.732 | 7232.601 | 3032.427 |
| TCGA-3A-A9IJ-01A-11R-A39D-07 | Alive | 1854 | 2480.649 | 1.596 | 1181.008 | 611.7835 | 5.8518 | 258.013 | 7629.206 | 1823.647 | 1035.244 | 1494.88 |
| TCGA-3A-A9IL-01A-11R-A38C-07 | Alive | 2741 | 84.7186 | 24.6649 | 614.478 | 821.4488 | 476.14 | 498.6602 | 5094.913 | 902.4141 | 182.8421 | 1718.501 |
| TCGA-3A-A9IN-01A-11R-A39D-07 | Alive | 2084 | 321.0162 | 2.8868 | 651.2702 | 882.2171 | 1.1547 | 984.4111 | 4366.628 | 542.1478 | 742.4942 | 1311.201 |
| TCGA-3A-A9IO-01A-11R-A38C-07 | Alive | 1942 | 241.5621 | 1.0345 | 678.1327 | 707.6167 | 667.2701 | 836.9326 | 7127.376 | 836.4154 | 3244.278 | 1477.822 |
| TCGA-3A-A9IR-01A-11R-A38C-07 | Alive | 1542 | 3839.2784 | 21.0004 | 543.5994 | 1146.069 | 0 | 2840.901 | 744.3077 | 1061.379 | 682.3395 | 2278.711 |
| TCGA-3A-A9IS-01A-21R-A39D-07 | Alive | 998 | 629.7138 | 0.4543 | 739.2095 | 875.0568 | 0 | 747.8419 | 8492.049 | 0.4543 | 6871.876 | 1918.673 |
| TCGA-3A-A9IU-01A-11R-A39D-07 | Dead | 458 | 10565.9051 | 8122.1441 | 6650.264 | 4664.763 | 42241.21 | 3547.452 | 11164.76 | 6041.74 | 6795.255 | 1656.415 |
| TCGA-3A-A9IV-01A-11R-A41B-07 | Alive | 1103 | 1913.8487 | 103.8305 | 806.5105 | 37.0422 | 92.6056 | 1484.496 | 1871.755 | 868.8088 | 185.7724 | 2793.882 |
| TCGA-3A-A9IX-01A-11R-A41B-07 | Alive | 1037 | 4224.196 | 2651.1485 | 2524.553 | 1152.425 | 13508.12 | 1049.923 | 3102.808 | 1181.828 | 3444.615 | 1369.27 |
| TCGA-3A-A9IZ-01A-12R-A41B-07 | Dead | 308 | 22159.8513 | 11875.8364 | 1473.606 | 1549.442 | 29722.68 | 6885.502 | 2387.361 | 2812.639 | 6677.323 | 1303.346 |
| TCGA-3A-A9J0-01A-11R-A41B-07 | Alive | 743 | 6383.417 | 30353.314 | 1817.798 | 4565.619 | 45580.67 | 3444.415 | 6304.199 | 4355.427 | 3553.208 | 2132.031 |
| TCGA-3E-AAAY-01A-11R-A38C-07 | Alive | 2285 | 4627.4988 | 5808.3862 | 2817.65 | 2923.94 | 27326.67 | 2137.494 | 2574.842 | 2375.427 | 3066.797 | 2218.918 |
| TCGA-3E-AAAZ-01A-11R-A38C-07 | Dead | 2182 | 6462.9768 | 7153.3283 | 2737.098 | 3380.703 | 24849.66 | 3190.726 | 4976.44 | 5779.731 | 6551.982 | 2792.072 |
| TCGA-F2-6879-01A-11R-2156-07 | Dead | 334 | 16960.8363 | 9237.927 | 3154.594 | 3983.216 | 61788.87 | 3521.79 | 3345.701 | 4303.887 | 9933.451 | 2484.099 |
| TCGA-F2-6880-01A-11R-2156-07 | Alive | 295 | 185.3329 | 44.1853 | 1501.074 | 1098.497 | 997.8521 | 1251.918 | 2005.523 | 1172.139 | 2728.444 | 1877.877 |
| TCGA-F2-7273-01A-11R-2156-07 | Dead | 592 | 1626.0812 | 865.7352 | 1069.328 | 601.1976 | 15165.4 | 1064.538 | 1868.53 | 917.8975 | 2842.582 | 1058.683 |
| TCGA-F2-7276-01A-11R-2156-07 | Dead | 216 | 1130.2746 | 581.3798 | 1427.328 | 825.5191 | 9214.669 | 1213.329 | 2149.364 | 992.2974 | 1803.751 | 1075.687 |
| TCGA-F2-A44G-01A-11R-A26U-07 | Dead | 233 | 10334.4371 | 15599.8896 | 2556.843 | 3946.468 | 51978.48 | 2459.625 | 4465.784 | 2578.918 | 7455.298 | 2588.3 |
| TCGA-F2-A44H-01A-11R-A26U-07 | Alive | 586 | 1223.7136 | 519.0157 | 1276.286 | 940.7159 | 6749.441 | 1546.98 | 1338.926 | 1866.89 | 3108.501 | 1587.248 |
| TCGA-F2-A7TX-01A-33R-A38C-07 | Dead | 95 | 4866.3149 | 8325.8575 | 911.6095 | 4248.461 | 23397.54 | 2585.312 | 4039.578 | 1913.808 | 5392.26 | 992.5242 |
| TCGA-F2-A8YN-01A-11R-A37L-07 | Alive | 517 | 23346.6722 | 24973.6055 | 1345.22 | 3628.798 | 80291.4 | 3479.443 | 4933.846 | 4677.369 | 3285.282 | 3194.64 |
| TCGA-FB-A4P5-01A-11R-A26U-07 | Dead | 179 | 5458.5074 | 8015.9797 | 1571.091 | 1932.086 | 14295.26 | 1105.146 | 2688.215 | 1424.369 | 1475.213 | 1303.795 |
| TCGA-FB-A4P6-01A-12R-A26U-07 | Alive | 767 | 2995.2316 | 3482.97 | 2416.894 | 1477.52 | 23394.41 | 2002.044 | 3504.087 | 1450.954 | 7888.965 | 1506.812 |
| TCGA-FB-A545-01A-11R-A26U-07 | Dead | 732 | 6487.5095 | 11168.8115 | 3424.678 | 3724.451 | 21686.6 | 4202.877 | 2793.338 | 2442.089 | 2847.843 | 2960.636 |
| TCGA-FB-A5VM-01A-11R-A32O-07 | Dead | 498 | 2541.471 | 3426.7084 | 1481.482 | 6406.364 | 6697.966 | 1864.893 | 3821.075 | 2210.746 | 1315.597 | 2221.701 |
| TCGA-FB-A78T-01A-12R-A32O-07 | Dead | 375 | 6891.3858 | 3936.7457 | 4044.112 | 5748.231 | 44384.94 | 4982.938 | 2071.161 | 5262.172 | 7878.069 | 2733.666 |
| TCGA-FB-A7DR-01A-21R-A33R-07 | Dead | 353 | 2834.3404 | 2768.6695 | 3807.092 | 1458.443 | 19662.98 | 2396.078 | 1597.993 | 1830.578 | 1677.802 | 1418.31 |
| TCGA-FB-AAPP-01A-12R-A41B-07 | Dead | 485 | 7271.7788 | 1110.7633 | 2066.423 | 2807.164 | 39464.44 | 3803.86 | 217.0057 | 3127.804 | 6853.069 | 2368.284 |
| TCGA-FB-AAPQ-01A-11R-A41B-07 | Dead | 1130 | 4856.948 | 3359.1198 | 1485.264 | 4982.59 | 40742.9 | 4833.615 | 1008.723 | 7682.091 | 4248.483 | 2897.835 |
| TCGA-FB-AAPS-01A-12R-A39D-07 | Alive | 228 | 2343.4483 | 4807.5862 | 691.0345 | 975.8621 | 7403.448 | 1029.655 | 888.2759 | 783.4483 | 980 | 460 |
| TCGA-FB-AAPU-01A-31R-A41B-07 | Dead | 381 | 11381.6712 | 3567.9891 | 6119.235 | 5340.532 | 69563.21 | 8210.659 | 1357.661 | 7044.742 | 7429.963 | 2990.908 |
| TCGA-FB-AAPY-01A-11R-A41B-07 | Dead | 1059 | 8942.5785 | 6526.5439 | 2293.066 | 3693.391 | 33003.79 | 2679.307 | 2887.866 | 3179.307 | 5052.546 | 2669.556 |
| TCGA-FB-AAPZ-01A-11R-A41B-07 | Alive | 716 | 7262.1289 | 3244.7502 | 1408.4 | 1876.901 | 41192.61 | 5328.023 | 4175.959 | 2110.065 | 4974.656 | 2689.356 |
| TCGA-FB-AAQ0-01A-31R-A41B-07 | Dead | 473 | 8569.1787 | 24239.7689 | 4139.826 | 8178.406 | 60322.33 | 6570.316 | 1998.863 | 5461.941 | 9900.767 | 2767.384 |
| TCGA-FB-AAQ1-01A-12R-A41B-07 | Dead | 123 | 7971.5214 | 11413.4399 | 3266.051 | 4317.262 | 1888.584 | 3846.615 | 6367.724 | 2676.493 | 5052.211 | 1634.774 |
| TCGA-FB-AAQ2-01A-31R-A41B-07 | Dead | 153 | 13845.5095 | 11187.5291 | 2994.881 | 3399.721 | 3011.633 | 2811.075 | 11233.6 | 4659.377 | 1909.26 | 3298.278 |
| TCGA-FB-AAQ3-01A-11R-A41B-07 | Dead | 31 | 13875.5235 | 13222.222 | 2036.499 | 3024.711 | 38558.31 | 2635.734 | 1890.82 | 4161.606 | 4603.898 | 3335.592 |
| TCGA-FB-AAQ6-01A-11R-A41B-07 | Dead | 244 | 5504.2254 | 12574.6479 | 2048.826 | 5328.639 | 29838.5 | 2221.296 | 3491.08 | 5740.845 | 8104.225 | 3820.657 |
| TCGA-H6-8124-01A-11R-2404-07 | Alive | 392 | 4125.3335 | 3329.1009 | 2708.756 | 2160.497 | 19428.41 | 15310.49 | 10214.68 | 1861.034 | 3307.269 | 1048.737 |
| TCGA-H6-A45N-01A-11R-A26U-07 | Dead | 421 | 5749.8051 | 3846.4536 | 2241.621 | 2500.39 | 43195.64 | 2316.446 | 2682.775 | 1692.128 | 3284.49 | 1544.817 |
| TCGA-H8-A6C1-01A-11R-A32O-07 | Alive | 671 | 4045.7974 | 2231.1422 | 3201.509 | 2721.983 | 21250 | 3751.616 | 1994.073 | 3752.155 | 5486.53 | 2469.289 |
| TCGA-HV-A5A3-01A-11R-A26U-07 | Dead | 128 | 17315.8757 | 9661.6326 | 4335.588 | 5875.78 | 59349.58 | 4347.268 | 10861.55 | 4807.457 | 9181.977 | 2230.865 |
| TCGA-HV-A5A4-01A-11R-A26U-07 | Alive | 232 | 9552.3912 | 9802.2569 | 3819.452 | 3950.027 | 47838.26 | 3347.663 | 2630.306 | 4447.072 | 8566.362 | 2235.895 |
| TCGA-HV-A5A5-01A-11R-A26U-07 | Alive | 289 | 3876.9497 | 2551.7042 | 2590.988 | 2083.767 | 36118.43 | 2506.066 | 2714.038 | 1762.565 | 3926.054 | 1797.805 |
| TCGA-HV-A5A6-01A-11R-A26U-07 | Dead | 2036 | 9039.6146 | 4951.2848 | 2115.632 | 3098.501 | 68691.65 | 6120.899 | 2404.711 | 3116.167 | 6125.268 | 2235.546 |
| TCGA-HV-A7OL-01A-11R-A33R-07 | Alive | 252 | 21205.1756 | 16812.6925 | 3382.625 | 7668.515 | 66873.69 | 4494.763 | 1872.458 | 6261.245 | 4812.076 | 3612.446 |
| TCGA-HV-A7OP-01A-11R-A33R-07 | Alive | 978 | 1670.0715 | 45326.8641 | 3617.978 | 6185.904 | 907.048 | 3059.244 | 1297.242 | 12266.6 | 5558.733 | 9133.81 |
| TCGA-HV-AA8V-01A-11R-A41B-07 | Alive | 920 | 8234.7236 | 7259.4568 | 2292.92 | 2312.318 | 25203.2 | 2264.307 | 2773.521 | 2430.65 | 2270.611 | 1650.34 |
| TCGA-HV-AA8X-01A-11R-A39D-07 | Dead | 532 | 9849.5786 | 11689.6477 | 4293.927 | 4138.319 | 76754.27 | 2994.165 | 3048.628 | 6312.946 | 5007.564 | 3224.552 |
| TCGA-HZ-7289-01A-11R-2156-07 | Dead | 661 | 13127.0256 | 4297.4386 | 2522.739 | 8116.048 | 51510.72 | 7596.445 | 720.3346 | 2603.241 | 6163.617 | 2851.542 |
| TCGA-HZ-7918-01A-11R-2156-07 | Alive | 969 | 729.6524 | 2.454 | 1600.409 | 422.4949 | 14572.6 | 1337.014 | 812.2699 | 1685.89 | 3539.468 | 1006.544 |
| TCGA-HZ-7919-01A-11R-2156-07 | Dead | 593 | 14424.4633 | 3724.5504 | 4794.027 | 2324.329 | 75520.87 | 4001.822 | 5644.062 | 3192.189 | 8740.791 | 2400.776 |
| TCGA-HZ-7920-01A-11R-2204-07 | Dead | 236 | 1526.2869 | 190.2374 | 820.8102 | 582.6216 | 18056.57 | 746.5329 | 3016.532 | 676.0166 | 1412.207 | 922.0403 |
| TCGA-HZ-7922-01A-11R-2156-07 | Alive | 4 | 14433.4047 | 2947.3233 | 1357.173 | 1773.019 | 76522.06 | 1957.602 | 3875.803 | 1480.942 | 9799.144 | 1330.621 |
| TCGA-HZ-7923-01A-12R-2156-07 | Alive | 314 | 2449.3042 | 1586.8125 | 867.1306 | 487.0775 | 32672.63 | 1177.601 | 2166.004 | 656.0636 | 4495.03 | 732.6044 |
| TCGA-HZ-7924-01A-11R-2156-07 | Alive | 840 | 7455.611 | 6634.1646 | 4049.377 | 2085.287 | 36620.7 | 2619.95 | 2951.87 | 2278.803 | 9135.412 | 2248.628 |
| TCGA-HZ-7925-01A-11R-2156-07 | Dead | 614 | 7409.1622 | 3256.0402 | 2001.569 | 1685.598 | 55491.69 | 1562.83 | 2982.742 | 2456.542 | 4202.071 | 1558.519 |
| TCGA-HZ-7926-01A-11R-2156-07 | Dead | 518 | 2957.9203 | 8680.3853 | 3996.936 | 2785.761 | 23277.82 | 6141.042 | 6502.03 | 2133.194 | 4230.623 | 2148.349 |
| TCGA-HZ-8001-01A-11R-2204-07 | Alive | 706 | 6964.8262 | 7203.681 | 2087.935 | 1542.331 | 34415.13 | 1776.278 | 4858.487 | 2020.45 | 1087.935 | 1523.517 |
| TCGA-HZ-8002-01A-11R-2204-07 | Dead | 366 | 1116.5775 | 829.59 | 1159.715 | 549.7326 | 18222.82 | 1170.054 | 1620.321 | 1017.469 | 4076.649 | 983.9572 |
| TCGA-HZ-8003-01A-21R-2204-07 | Dead | 596 | 854.2213 | 792.8684 | 1536.445 | 790.2465 | 34664.39 | 1070.792 | 2153.645 | 990.5611 | 2453.592 | 1012.585 |
| TCGA-HZ-8005-01A-11R-2204-07 | Dead | 120 | 4126.3476 | 8225.097 | 1124.192 | 5468.305 | 206.1233 | 2355.757 | 8704.614 | 1177.232 | 4927.555 | 1871.928 |
| TCGA-HZ-8315-01A-11R-2404-07 | Dead | 299 | 9707.3078 | 4147.6713 | 2540.704 | 1288.527 | 80393.03 | 2483.529 | 3279.061 | 1888.3 | 6307.081 | 1518.364 |
| TCGA-HZ-8317-01A-11R-2404-07 | Dead | 378 | 4087.6768 | 2687.3965 | 1726.01 | 1388.556 | 29713.78 | 1936.536 | 1477.762 | 1746.4 | 3952.593 | 1415.063 |
| TCGA-HZ-8519-01A-11R-2404-07 | Alive | 454 | 1264.397 | 1935.3499 | 929.8432 | 800.1345 | 16744.03 | 615.5894 | 3850.4 | 1612.396 | 1810.123 | 1033.188 |
| TCGA-HZ-8636-01A-21R-2404-07 | Dead | 545 | 7282.7056 | 6146.2903 | 3026.479 | 2097.702 | 42878.27 | 3230.971 | 4603.338 | 2846.456 | 3354.016 | 1870.489 |
| TCGA-HZ-8637-01A-11R-2404-07 | Dead | 517 | 1252.9619 | 2377.8418 | 1958.694 | 580.8517 | 1144.412 | 1165.226 | 1103.746 | 1409.862 | 2323.087 | 614.1531 |
| TCGA-HZ-8638-01A-11R-2404-07 | Dead | 151 | 10006.6424 | 3639.728 | 2901.787 | 1666.298 | 63631.19 | 2363.435 | 1720.07 | 1998.102 | 8565.871 | 1946.544 |
| TCGA-HZ-A49G-01A-11R-A26U-07 | Alive | 660 | 5344.0919 | 8104.4858 | 2153.72 | 1835.339 | 42909.74 | 2060.175 | 2518.6 | 1655.908 | 2858.315 | 1445.295 |
| TCGA-HZ-A49H-01A-11R-A26U-07 | Alive | 491 | 4523.6908 | 249.3766 | 1046.827 | 1380.992 | 3059.573 | 1547.797 | 955.3893 | 1976.171 | 3185.37 | 1294.541 |
| TCGA-HZ-A49I-01A-12R-A26U-07 | Dead | 308 | 6599.3673 | 15274.3777 | 3793.701 | 3680.924 | 7651.767 | 3002.063 | 4167.79 | 1886.398 | 3495.53 | 2214.276 |
| TCGA-HZ-A4BH-01A-11R-A26U-07 | Alive | 194 | 6688.7449 | 5742.2606 | 4016.799 | 1575.234 | 38816.41 | 3986.921 | 2816.895 | 3009.359 | 4634.989 | 2500.12 |
| TCGA-HZ-A4BK-01A-11R-A26U-07 | Alive | 657 | 7117.1649 | 4153.3269 | 2735.776 | 3207.811 | 52664.9 | 2844.634 | 2492.285 | 2241.08 | 7760.849 | 2117.165 |
| TCGA-HZ-A77O-01A-11R-A33R-07 | Dead | 160 | 2722.4441 | 5180.8366 | 4801.643 | 5943.652 | 8585.829 | 2904.014 | 2796.622 | 2457.839 | 2324.429 | 3511.278 |
| TCGA-HZ-A77P-01A-11R-A33R-07 | Alive | 330 | 3063.6503 | 3134.5859 | 1080.138 | 1307.132 | 15256.14 | 1215.491 | 2032.976 | 1213.957 | 3282.592 | 1063.267 |
| TCGA-HZ-A77Q-01A-11R-A36G-07 | Alive | 33 | 7425.3819 | 4814.3361 | 1040.541 | 1206.816 | 21137.49 | 1538.778 | 1720.329 | 1463.572 | 3199.177 | 1091.069 |
| TCGA-HZ-A8P0-01A-11R-A36G-07 | Alive | 0 | 3203.532 | 36103.8987 | 2648.333 | 3061.242 | 4424.97 | 3330.53 | 1694.188 | 4557.287 | 3508.06 | 2837.167 |
| TCGA-HZ-A8P1-01A-11R-A37L-07 | Alive | 7 | 9025.7485 | 2761.0778 | 4161.677 | 5653.293 | 73257.49 | 4468.264 | 682.0359 | 5822.755 | 7143.114 | 3226.946 |
| TCGA-HZ-A9TJ-01A-11R-A41I-07 | Alive | 603 | 10173.3164 | 7780.3361 | 3146.729 | 4114.116 | 29030.78 | 8304.409 | 3944.007 | 5170.281 | 7062.098 | 2333.986 |
| TCGA-IB-7644-01A-11R-2156-07 | Dead | 394 | 11418.0982 | 10438.3436 | 3985.276 | 2651.841 | 57585.28 | 2705.522 | 3617.791 | 4092.945 | 17470.25 | 2975.767 |
| TCGA-IB-7645-01A-22R-2204-07 | Dead | 1502 | 2924.7401 | 1508.5239 | 3525.988 | 439.0852 | 55882.33 | 1797.089 | 2095.218 | 1675.26 | 2465.697 | 1236.175 |
| TCGA-IB-7646-01A-11R-2156-07 | Dead | 145 | 6316.2437 | 1592.8934 | 4019.797 | 1267.005 | 33606.09 | 1487.817 | 9021.32 | 1938.579 | 2505.076 | 806.599 |
| TCGA-IB-7647-01A-11R-2156-07 | Dead | 666 | 5675.379 | 931.7825 | 2166.231 | 2903.032 | 67267.14 | 2845.008 | 2403.032 | 1404.077 | 6865.656 | 1458.181 |
| TCGA-IB-7649-01A-11R-2156-07 | Dead | 467 | 3953.71 | 1321.9877 | 2027.91 | 1599.728 | 30190.61 | 1849.558 | 2387.338 | 2231.45 | 4482.641 | 1665.078 |
| TCGA-IB-7651-01A-11R-2156-07 | Dead | 603 | 4153.4075 | 3646.7554 | 2700.516 | 2288.352 | 10475.43 | 2166.106 | 2569.916 | 1339.126 | 4368.993 | 986.9671 |
| TCGA-IB-7652-01A-11R-2156-07 | Alive | 1116 | 4080.3074 | 7745.2955 | 1992.844 | 563.7424 | 41829.31 | 1918.632 | 4699.178 | 2097.27 | 3738.405 | 1883.912 |
| TCGA-IB-7654-01A-11R-2156-07 | Dead | 476 | 6085.9448 | 5383.8039 | 3601.613 | 2158.238 | 7915.917 | 1984.176 | 3835.247 | 3647.844 | 10282.97 | 2985.107 |
| TCGA-IB-7885-01A-11R-2156-07 | Alive | 1257 | 7104.8083 | 9144.919 | 3599.228 | 1492.993 | 11870.77 | 2491.567 | 3823.11 | 2371.067 | 3276.664 | 1219.434 |
| TCGA-IB-7886-01A-11R-2156-07 | Dead | 123 | 9323.9997 | 1223.5607 | 1688.047 | 1382.533 | 59847.11 | 3228.799 | 3687.634 | 3416.025 | 7886.843 | 1949.485 |
| TCGA-IB-7887-01A-11R-2156-07 | Dead | 110 | 9452.5707 | 14636.4194 | 2376.807 | 844.0046 | 26864.15 | 2063.589 | 5785.408 | 3174.564 | 4650.609 | 2649.032 |
| TCGA-IB-7888-01A-11R-2156-07 | Dead | 1332 | 4367.0886 | 466.7034 | 1386.902 | 868.4645 | 26821.68 | 1346.494 | 1359.384 | 2139.241 | 3166.208 | 1239.956 |
| TCGA-IB-7889-01A-11R-2156-07 | Dead | 481 | 6622.5793 | 4629.9959 | 3405.027 | 1513.803 | 38755.67 | 3483.313 | 5112.897 | 4379.481 | 4618.047 | 3058.508 |
| TCGA-IB-7890-01A-12R-2204-07 | Dead | 598 | 2278.83 | 5936.1292 | 3106.936 | 1779.711 | 41688.7 | 2051.818 | 7817.304 | 1443.037 | 1638.79 | 1435.35 |
| TCGA-IB-7891-01A-11R-2204-07 | Dead | 913 | 2468.1034 | 263.3215 | 2027.662 | 749.6515 | 38553.88 | 1674.279 | 2532.004 | 2023.373 | 4819.556 | 1401.523 |
| TCGA-IB-7893-01A-11R-2204-07 | Dead | 117 | 3058.8456 | 3312.9685 | 2162.669 | 1908.921 | 6624.813 | 2354.948 | 1932.534 | 2256.372 | 1697.151 | 1480.135 |
| TCGA-IB-7897-01A-21R-2204-07 | Dead | 486 | 757.8605 | 207.8063 | 975.4246 | 380.918 | 1554.752 | 646.5486 | 3668.956 | 593.7839 | 2265.631 | 767.6184 |
| TCGA-IB-8126-01A-11R-2404-07 | Alive | 462 | 671.7488 | 228.4907 | 1362.71 | 548.9265 | 2584.072 | 966.1106 | 1072.465 | 897.4948 | 3771.125 | 909.8457 |
| TCGA-IB-8127-01A-11R-2404-07 | Alive | 522 | 8604.5457 | 4412.3551 | 2484.491 | 1941.074 | 42632.65 | 3112.797 | 5404.908 | 2331.93 | 9695.007 | 1508.774 |
| TCGA-IB-A5SO-01A-11R-A32O-07 | Dead | 365 | 6354.9012 | 2942.2704 | 1907.788 | 2364.587 | 74673.77 | 3858.969 | 2917.086 | 2183.65 | 5027.509 | 1481.596 |
| TCGA-IB-A5SP-01A-11R-A32O-07 | Alive | 482 | 17861.0771 | 11939.8742 | 3139.801 | 6774.051 | 113957.8 | 3119.82 | 5156.178 | 4761.228 | 6087.204 | 3622.066 |
| TCGA-IB-A5SQ-01A-11R-A32O-07 | Dead | 219 | 5667.1054 | 4852.4773 | 1352.322 | 1572.889 | 15908.77 | 1608.67 | 2912.957 | 2377.714 | 1387.613 | 1211.649 |
| TCGA-IB-A5SS-01A-11R-A32O-07 | Dead | 460 | 6279.1536 | 4094.8857 | 2357.339 | 3406.016 | 18866.69 | 2448.05 | 8583.357 | 2435.525 | 4714.679 | 2064.333 |
| TCGA-IB-A5ST-01A-11R-A32O-07 | Alive | 635 | 4642.2018 | 3603.7571 | 1555.264 | 915.6837 | 33363.91 | 1043.687 | 781.564 | 1175.623 | 3158.148 | 598.9515 |
| TCGA-IB-A6UF-01A-23R-A33R-07 | Alive | 666 | 5954.5435 | 19757.2504 | 2126.347 | 5431.75 | 6615.624 | 2500.735 | 8852.312 | 2889.096 | 4042.132 | 1148.218 |
| TCGA-IB-A6UG-01A-32R-A33R-07 | Dead | 41 | 2812.248 | 6413.5787 | 2467.628 | 4791.933 | 8087.293 | 2318.4 | 4247.09 | 2278.141 | 3905.691 | 1687.134 |
| TCGA-IB-A7LX-01A-12R-A36G-07 | Dead | 250 | 3547.8449 | 4216.4525 | 4274.772 | 4756.941 | 26238.72 | 6414.224 | 31532.01 | 4159.511 | 5504.532 | 2575.241 |
| TCGA-IB-A7M4-01A-11R-A36G-07 | Alive | 483 | 7822.9774 | 6773.0155 | 2388.029 | 6932.285 | 42111.08 | 2604.986 | 8529.546 | 3498.859 | 4787.725 | 2472.737 |
| TCGA-IB-AAUM-01A-11R-A37L-07 | Alive | 8 | 2781.362 | 3093.9068 | 2028.674 | 1306.093 | 11113.26 | 1751.255 | 1696.774 | 2362.007 | 4683.871 | 1563.441 |
| TCGA-IB-AAUN-01A-12R-A38C-07 | Dead | 144 | 3026.4193 | 8055.6492 | 2446.318 | 4149.522 | 31349.63 | 3069.14 | 12269.25 | 2183.249 | 2383.924 | 2570.545 |
| TCGA-IB-AAUO-01A-12R-A38C-07 | Dead | 239 | 10993.4433 | 16062.2322 | 4395.902 | 5732.822 | 12206.41 | 3055.538 | 5871.04 | 3234.581 | 5128.306 | 2570.547 |
| TCGA-IB-AAUP-01A-11R-A37L-07 | Alive | 431 | 4647.2233 | 6778.9973 | 1043.667 | 1129.026 | 5954.007 | 972.3881 | 1902.097 | 728.6311 | 2037.615 | 1009.788 |
| TCGA-IB-AAUQ-01A-22R-A41I-07 | Dead | 183 | 8866.0714 | 12477.6786 | 1622.449 | 2672.194 | 27112.24 | 2179.847 | 4646.684 | 1644.133 | 2415.179 | 1375 |
| TCGA-IB-AAUR-01A-21R-A38C-07 | Alive | 338 | 3834.0848 | 2096.0325 | 832.2813 | 1052.75 | 11757.89 | 767.8088 | 1022.092 | 1445.897 | 1501.803 | 750.2254 |
| TCGA-IB-AAUS-01A-12R-A38C-07 | Alive | 225 | 2318.4713 | 1722.685 | 901.0289 | 904.9486 | 16245.96 | 879.4708 | 1379.226 | 770.2107 | 1536.012 | 712.3959 |
| TCGA-IB-AAUT-01A-11R-A37L-07 | Alive | 287 | 1044.2584 | 2761.3636 | 1135.168 | 1285.287 | 13424.64 | 1496.412 | 1995.813 | 1366.029 | 4802.632 | 1438.995 |
| TCGA-IB-AAUU-01A-11R-A37L-07 | Alive | 245 | 9229.2288 | 11109.9276 | 4666.809 | 6045.164 | 67373.67 | 4558.044 | 5185.769 | 4379.208 | 5964.21 | 2542.821 |
| TCGA-IB-AAUV-01A-11R-A38C-07 | Alive | 404 | 126.7081 | 36.853 | 273.2919 | 293.5818 | 1110.145 | 411.1801 | 807.0393 | 242.236 | 412.4224 | 255.9006 |
| TCGA-IB-AAUW-01A-12R-A38C-07 | Dead | 230 | 4394.0228 | 1779.7877 | 2177.743 | 1397.169 | 19383.01 | 1735.745 | 3595.753 | 1559.182 | 5710.185 | 1590.248 |
| TCGA-L1-A7W4-01A-12R-A36G-07 | Dead | 278 | 9107.3374 | 5825.0561 | 432.7673 | 3841.504 | 68.3542 | 3374.987 | 5593.934 | 3336.537 | 3841.931 | 1023.176 |
| TCGA-LB-A7SX-01A-11R-A33R-07 | Dead | 393 | 3073.6403 | 11334.8023 | 2632.958 | 1979.358 | 37218.6 | 1664.386 | 6060.536 | 3229.039 | 6724.342 | 2543.43 |
| TCGA-LB-A8F3-01A-11R-A36G-07 | Alive | 379 | 193.9086 | 1.0152 | 3698.477 | 3205.584 | 2304.569 | 2268.02 | 2292.386 | 6230.965 | 5761.421 | 2917.259 |
| TCGA-LB-A9Q5-01A-11R-A39D-07 | Dead | 313 | 5828.728 | 11713.1765 | 4330.669 | 2640.786 | 40973.74 | 1861.612 | 4827.586 | 1685.316 | 3118.52 | 1855.218 |
| TCGA-M8-A5N4-01A-11R-A26U-07 | Alive | 584 | 9657.0936 | 17398.6215 | 3947.157 | 4727.743 | 58940.26 | 4401.493 | 3180.931 | 2762.78 | 3789.202 | 2020.678 |
| TCGA-OE-A75W-01A-12R-A32O-07 | Dead | 267 | 7721.9464 | 19127.1102 | 2146.475 | 7207.547 | 44478.65 | 6806.356 | 1925.025 | 4505.958 | 4090.864 | 3340.616 |
| TCGA-PZ-A5RE-01A-11R-A32O-07 | Dead | 470 | 19880.5985 | 7525.6759 | 4098.242 | 4868.003 | 64522.17 | 19055.59 | 6813.637 | 3897.453 | 5159.91 | 2504.298 |
| TCGA-Q3-A5QY-01A-12R-A32O-07 | Alive | 416 | 4459.4659 | 3477.1602 | 629.6968 | 983.3694 | 7350.54 | 649.3748 | 895.6161 | 910.5075 | 831.7954 | 342.504 |
| TCGA-Q3-AA2A-01A-11R-A37L-07 | Alive | 95 | 8866.6186 | 9878.1543 | 1751.262 | 4715.213 | 32257.39 | 4634.463 | 2191.781 | 3306.417 | 7068.493 | 2248.738 |
| TCGA-RB-A7B8-01A-12R-A33R-07 | Dead | 466 | 12188.7694 | 12462.3656 | 2986.261 | 3636.201 | 23539.43 | 3318.399 | 1909.2 | 3752.688 | 3887.694 | 2159.498 |
| TCGA-RB-AA9M-01A-11R-A39D-07 | Alive | 286 | 3869.0987 | 5845.4936 | 3350.215 | 2778.541 | 64080.26 | 2229.185 | 5278.112 | 2332.618 | 4272.532 | 1871.245 |
| TCGA-RL-AAAS-01A-32R-A39D-07 | Alive | 9 | 2253.4834 | 2873.5263 | 1392.819 | 1197.213 | 17294.75 | 1651.661 | 1973.205 | 1571.811 | 2756.163 | 1739.014 |
| TCGA-S4-A8RM-01A-11R-A37L-07 | Alive | 737 | 5518.5031 | 5330.9771 | 7954.262 | 3648.649 | 66677.34 | 6811.642 | 2526.819 | 4470.27 | 9223.285 | 2742.62 |
| TCGA-S4-A8RO-01A-12R-A37L-07 | Alive | 525 | 6553.2353 | 2855.7885 | 3079.189 | 2771.068 | 28304.73 | 3095.83 | 1679.787 | 4732.752 | 2816.454 | 2495.726 |
| TCGA-S4-A8RP-01A-11R-A36G-07 | Dead | 702 | 8699.8412 | 6157.226 | 3467.443 | 1609.847 | 30060.88 | 2705.474 | 3363.155 | 2946.533 | 5605.082 | 1838.01 |
| TCGA-US-A774-01A-21R-A32O-07 | Dead | 695 | 4908.6316 | 5157.0526 | 2529.263 | 2075.79 | 33660.21 | 1860.632 | 3280.842 | 2103.579 | 3804.211 | 1628.211 |
| TCGA-US-A776-01A-13R-A33R-07 | Alive | 1216 | 14023.5179 | 29.8873 | 2046.056 | 3618.814 | 9144.537 | 4005.88 | 545.3209 | 3164.135 | 8166.095 | 2899.559 |
| TCGA-US-A779-01A-11R-A32O-07 | Dead | 511 | 14503.786 | 24622.5308 | 6836.009 | 6475.795 | 44702.33 | 4896.898 | 5997.693 | 5415.304 | 8329.261 | 4268.161 |
| TCGA-US-A77E-01A-11R-A32O-07 | Dead | 430 | 8757.2052 | 21660.6987 | 2031.004 | 3589.956 | 40683.84 | 3327.074 | 2482.969 | 2308.297 | 7537.555 | 2056.769 |
| TCGA-US-A77G-01A-11R-A32O-07 | Dead | 12 | 13508.9 | 16839.3428 | 6678.229 | 5053.4 | 26460.52 | 5231.671 | 4536.741 | 4704.245 | 10557.28 | 3093.108 |
| TCGA-US-A77J-01A-11R-A32O-07 | Dead | 568 | 1174.1778 | 1096.8331 | 528.0146 | 1006.09 | 18048.11 | 1516.443 | 1904.994 | 903.1669 | 2030.451 | 1084.044 |
| TCGA-XD-AAUG-01A-61R-A41B-07 | Alive | 420 | 1008.1081 | 1592.5676 | 510.1351 | 871.6216 | 2891.216 | 954.7297 | 2278.378 | 755.4054 | 1391.892 | 808.7838 |
| TCGA-XD-AAUH-01A-42R-A41B-07 | Alive | 395 | 2132.7434 | 1969.8074 | 1865.695 | 982.8214 | 21992.19 | 1352.421 | 3612.181 | 2074.44 | 1333.68 | 937.5325 |
| TCGA-XD-AAUI-01A-42R-A41B-07 | Dead | 366 | 2733.5315 | 1549.7771 | 1766.716 | 4006.439 | 9545.32 | 3035.661 | 2412.581 | 3143.14 | 2648.836 | 2329.371 |
| TCGA-XD-AAUL-01A-21R-A39D-07 | Alive | 498 | 12469.8545 | 6239.0852 | 3700.208 | 3533.472 | 26116.01 | 5249.48 | 5381.289 | 3210.811 | 5702.703 | 1902.287 |
| TCGA-XN-A8T3-01A-11R-A36G-07 | Alive | 951 | 3072.7848 | 375 | 1008.439 | 2301.16 | 2327.532 | 1854.43 | 2731.54 | 1646.097 | 2727.321 | 1198.84 |
| TCGA-XN-A8T5-01A-12R-A36G-07 | Alive | 720 | 3971.3945 | 11481.5256 | 1055.423 | 1228.844 | 18594.76 | 1173.713 | 3865.316 | 823.5995 | 2235.995 | 994.0405 |
| TCGA-YB-A89D-01A-12R-A36G-07 | Alive | 350 | 6866.3926 | 10015.4162 | 2024.152 | 2899.281 | 50475.33 | 3105.858 | 3560.123 | 2118.191 | 3011.819 | 2065.776 |
| TCGA-YH-A8SY-01A-11R-A37L-07 | Alive | 388 | 5358.0607 | 13773.3645 | 3067.757 | 3613.902 | 22248.83 | 3257.009 | 2328.271 | 2367.991 | 1739.486 | 904.7897 |
| TCGA-YY-A8LH-01A-11R-A36G-07 | Alive | 2016 | 11767.6111 | 17562.3415 | 3996.031 | 7087.642 | 36187.85 | 6487.488 | 2504.685 | 5362.584 | 6020.505 | 3372.947 |
| TCGA-Z5-AAPL-01A-12R-A41B-07 | Alive | 467 | 2634.7578 | 1046.1538 | 643.8746 | 943.5897 | 19178.35 | 662.1083 | 480.9117 | 960.6838 | 1657.55 | 900.2849 |
